# Paracrine Interactions between Adipocytes and Tumor Cells Recruit and Modify Macrophages to the Mammary Tumor Microenvironment: The Role of Obesity and Inflammation in Breast Adipose Tissue 

**DOI:** 10.3390/cancers7010143

**Published:** 2015-01-15

**Authors:** Ana M. Santander, Omar Lopez-Ocejo, Olivia Casas, Thais Agostini, Lidia Sanchez, Eduardo Lamas-Basulto, Roberto Carrio, Margot P. Cleary, Ruben R. Gonzalez-Perez, Marta Torroella-Kouri

**Affiliations:** 1 Department of Microbiology and Immunology, University of Miami Miller School of Medicine, 1600 NW 10th Ave, Miami, FL 33136, USA; E-Mails: AMsantander@med.miami.edu (A.M.S.); olopez2@med.miami.edu (O.L.-O.); o.casas1@umiami.edu (O.C.); Thais.Agostini@treetop.com (T.A.); lgs20@miami.edu (L.S.); e.lamasbasulto@umiami.edu (E.L.-B.); rcarrio@med.miami.edu (R.C.); 2 Hormel Institute, University of Minnesota, Austin, MN 55912, USA; E-Mail: mpcleary@hi.umn.edu; 3 Department of Microbiology, Biochemistry and Immunology, Morehouse School of Medicine, Atlanta, GA 30314, USA; E-Mail: rgonzalez@msm.edu; 4 Sylvester Comprehensive Cancer Center, University of Miami Miller School of Medicine, 1475 NW 12th Ave, Miami, FL 33136, USA

**Keywords:** breast cancer, obesity, breast adipose tissue, inflammation, tumor microenvironment, macrophages, adipocytes, breast tumor cells, chemotaxis, diet-induced obesity, tumor progression

## Abstract

The relationship between obesity and breast cancer (BC) has focused on serum factors. However, the mammary gland contains adipose tissue (AT) which may enable the crosstalk between adipocytes and tumor cells contributing to tumor macrophage recruitment. We hypothesize that the breast AT (bAT) is inflamed in obese females and plays a major role in breast cancer development. The effects of this interplay on macrophage chemotaxis were examined *in vitro*, using co-cultures of mouse macrophages, mammary tumor cells and adipocytes. Macrophages were exposed to the adipocyte and tumor paracrine factors leptin, CCL2 and lauric acid (alone or in combinations). In cell supernatants Luminex identified additional molecules with chemotactic and other pro-tumor functions. Focus on the adipokine leptin, which has been shown to have a central role in breast cancer pathogenesis, indicated it modulates macrophage phenotypes and functions. *In vivo* experiments demonstrate that mammary tumors from obese mice are larger and that bAT from obese tumor-bearers contains higher numbers of macrophages/CLS and hypertrophic adipocytes than bAT from lean tumor-bearers, thus confirming it is more inflamed. Also, bAT distal from the tumor is more inflamed in obese than in lean mice. Our results reveal that bAT plays a role in breast cancer development in obesity.

## 1. Introduction

Obesity has been recently recognized as a new disease and one of the leading health problems in the United States [1,2], associated with increased risk of cardiovascular disorders, diabetes and cancer [3]. Breast cancer, the most common cancer in women and the second leading cause of mortality from this disease in our country, is a more prevailing and aggressive disease in overweight and obese women [4]. Obesity increases the risk of developing ER^+^ postmenopausal breast cancer, although there are contradictory evidences that obesity [5] or rather circulatory estrogen [6] protects against premenopausal breast cancer, while there are also indications that it is associated with poor breast cancer prognosis regardless of menopausal status [7]. However, the causes of these associations are not completely understood. Chronic inflammation is common to obesity [8] and to many cancers [9], and elevated numbers of macrophages colonize obese adipose tissues [10] and also advanced cancers [11,12]. Macrophages, central cells in the inflammatory response, are also the most abundant immune cell type in tumor microenvironments, where they help tumor progression and are a sign of poor tumor prognosis [13,14,15]. In previous studies we have shown that blood monocytes and peripheral and tumor-associated macrophages (TAMs) from mammary tumor-bearing mice are impacted to different degrees in their phenotypes and functions by the tumors and the factors they secrete [16,17,18,19,20].

Most studies linking obesity and cancer have focused on the systemic inflammatory effects of adiposity [21], which result in elevated circulating levels of pro-inflammatory adipokines, cytokines, chemokines, insulin, estrogen and other factors directly secreted by obese adipose tissues or indirectly induced to be secreted by other tissues. Given that the mammary gland contains a significant amount of white adipose tissue required for normal breast gland development, it is plausible that this local adipose tissue in the breast (bAT) could be inflamed in obese females as the visceral fat is, and may thus play a role in breast cancer development in conditions of obesity. The local role of bAT inflammation on the association of breast cancer with obesity has been recently analyzed in mice and humans [22,23], but it is still insufficiently understood and requires further examination. Breast cancer is one of the cancers not currently associated with chronic inflammation despite the association of this disease with obesity. Whether obesity also promotes breast cancer through its effect in local adipose tissue inflammation and innate immune signaling in the breast, where cancer occurs, has not received enough attention and should be more thoroughly investigated. 

We propose that obesity impacts breast cancer not only systemically but also at the local level in the breast. We hypothesize that mammary paracrine factors secreted by adipocytes and tumor cells in the breast of obese/overweight females modulate macrophage recruitment to the tumor microenvironment, as well as macrophage phenotypes, activation patterns and functions, contributing to breast cancer progression. Moreover, paracrine factors resulting from the crosstalk among adipocytes, tumor cells and macrophages in the breast tumor microenvironment might contribute to tumor progression via additional mechanisms. 

To analyze how adipocyte/tumor cell-derived factors in the breast affect macrophage recruitment/functions and promote breast cancer progression, we used a reductionist *in vitro* co-culture setting to mimic the mammary tumor microenvironment in obese mice. These *in vitro* studies were complemented with *in vivo* mouse experiments using diet-induced obese (DIO) mammary tumor-bearing female mice in which tumor progression and bAT inflammation were assessed. We examined the overarching hypothesis that bAT in obese mice exhibits inflammatory and tumor-promoting capabilities that foster breast cancer development. For the *in vitro* studies, we investigated the crosstalk between three critical cell types in the breast cancer microenvironment, *i.e.*, the mammary tumor cell, the adipocyte and the macrophage. We interrogated whether adipocytes and mammary tumor cells, acting together or independently, recruit macrophages and modify their activation profiles/functions, to further tumor progression. Specifically, we analyzed whether adipocytes’ crosstalk with tumor cells and macrophages modulates their mutual production of paracrine factors such as the pro-tumor adipokine leptin, the pro-inflammatory saturated free fatty acid (FFA) lauric acid, and the chemokine CCL2, thus contributing to macrophage recruitment and tumor progression. In addition, we revealed the existence of numerous other proteins with chemotactic, proinflammatory and tumor-promoting properties as a result of the interplay between those three cell types. Furthermore we show the effects of leptin in the modulation of macrophage phenotypes and functions. Overall, our results indicate that local breast adipocytes are important players of tumor progression in the mammary cancer microenvironment of obese mice, and that their crosstalk with mammary tumor cells and macrophages is critical in the production of different paracrine factors that contribute to macrophage chemotaxis/function and tumor development. We also demonstrate that mammary tumors in obese mice are larger than those in lean ones, and that the bAT from obese tumor-bearers is inflamed, containing higher numbers of macrophages, crown-like structures (CLS) and hypertrophic adipocytes than the bAT from lean tumor-bearers. Our study also reveals that bAT distant from the tumor site is less inflamed in obese females that the bAT in the tumor microenvironment, suggesting that the contact of adipose tissue with tumor cells contributes to macrophage recruitment. Collectively, we conclude that obesity plays not only a systemic but also a local role in breast cancer development through the increased presence of inflammatory cells and molecules in the bAT.

## 2. Experimental Section 

### 2.1. Materials 

Recombinant (murine) and synthetic paracrine factors were from the following sources: leptin and CCL2 were from Peprotech (Rocky Hill, NJ, USA) and the saturated FFA lauric acid was from Nu-Chek Prep, Inc. (Elysian, MN, USA). Stock solutions were prepared using the following solvents: sterile water (leptin); PBS+0.1%BSA (CCL2) and 100% ethanol (lauric acid). Further dilutions were prepared in culture media to add to the cell cultures. Specific paracrine factor inhibitors were the following: CCL2 blocking antibody was from R & D System (Minneapolis, MN, USA); pegylated leptin receptor blocking peptide (LPrA2) was provided by Dr. R. R Gonzalez-Perez, whereas Eritoran, a TLR4 antagonist, was kindly supplied by Eisai Inc. (Andover, MA, USA). Matrigel was purchased from BD Biosciences (San Jose, CA, USA). 

### 2.2. Cells

E0771 mammary tumor cells (syngeneic to C57BL6 mice), a very aggressive ER^+^ and estrogen-dependent breast cancer cell line that metastasizes to the peritoneal cavity and lungs and closely resembles aggressive forms of human breast cancer [24], was generously provided by Dr. M. Kolonin (Center for Stem Cell Research, Institute of Molecular Medicine, University of Texas, Houston, TX, USA). The human acute monocytic leukemia THP-1 cell line was kindly supplied by Dr. V. Gupta (Rush University, Chicago, IL, USA). Adipocytes were either *in vitro* differentiated from 3T3-L1 murine preadipocytes using the 3T3-L1 Growth and Differentiation Feeding Schedule following instructions from ZenBio Inc. (Research Triangle Park, NC, USA), or *ex vivo* isolated from visceral fat of diet-induced obese (DIO) C57BL6 female mice. Peritoneal elicited macrophages (N-PEMs) and tumor-associated macrophages (TAMs) were isolated from C57BL6 normal and tumor-bearing female mice, respectively, as previously described [17,20].

### 2.3. Ex vivo Isolation of Adipocytes 

Adipocytes were separated from other cell types present in the visceral white adipose tissue of diet-induced obese (DIO) female mice by enzymatic digestion of the tissue with collagenase. Briefly, approximately 200–400 mg of tissue were minced into small pieces (~1 mm) and incubated in 4 volumes of 1 mg/mL collagenase IV (Worthington Biochemical Corporation, Lakewood, NJ, USA) in PBS for 30 min at 37 °C. The sample was centrifuged at 600 × g for 2 min to obtain an adipocyte fraction that floats and the stromal vascular fraction (SVF) that pellets. *Ex-vivo* isolated adipocytes were then cultured in the ZenBio’s adipocyte medium AM-1-L1 (ZenBio Inc.), or they were mixed with macrophages and tumor cells in co-cultures as described below.

### 2.4. Pre-Treatment of Macrophages with Conditioned Medium from E0771 Cells, Adipocytes, Their Co-Cultures, and with Recombinant Paracrine Factors

Conditioned media from adipocytes (*in vitro* differentiated or *ex vivo* isolated), from E0771 mammary tumor cells and from their co-cultures without and with peritoneal elicited macrophages from normal mice (N-PEMs) were centrifuged and supernatants were frozen at −80 °C for further pre-treatment of N-PEMs and for protein analysis by ELISA and Luminex. Recombinant or synthetic paracrine factors (leptin, CCL2 and lauric acid) were prepared fresh just before use. N-PEMs were isolated and adhered to plastic tissue culture dishes, cultured in Nutridoma serum-free culture medium (Roche) and pre-treated for the referred time intervals in the different experimental conditions explained in the figures. Macrophages were lysed and Western blot analysis was performed (as described below) or supernatants were collected for ELISA or Luminex studies. Viability was assessed by trypan blue exclusion. 

### 2.5. Cell Co-Cultures

We co-cultured mouse peritoneal elicited macrophages (N-PEMs), adipocytes (*in vitro* differentiated or *ex vivo* isolated) and E0771 mammary tumor cells for 48 h. To do this, when 3T3-L1 *in vitro* differentiated adipocytes were used, first 3T3-L1 fibroblasts were grown until 80%–90% confluence, at which point they started *in vitro* differentiation into adipocytes for 11 days following instructions from ZenBio Inc.; 5 × 10^5^ macrophages and 5 × 10^5^ E0771 cells per well were then plated onto those 3T3-L1 differentiated adipocytes. On the other hand, when *ex vivo* isolated adipocytes were used, the floating fraction of adipocytes was isolated from visceral fat as described above, and 5 × 10^5^ adipocytes from this fraction were mixed with 5 × 10^5^ macrophages and 5 × 10^5^ E0771 cells and seeded altogether. Co-cultures were carried out in 6-well plates, and conditioned medium was harvested, centrifuged and supernatants were frozen at −80 °C for further analyses. By setting up the co-cultures using these cell numbers, at the end of the 48 h of co-cultures, the numbers of E0771 tumor cells, which do proliferate, will exceed the numbers of macrophages and adipocytes, as is the case in the mammary tumor microenvironment.

### 2.6. Migration (Chemotaxis) Assay

Assays were done in triplicate in migration chambers [24 well cell culture plate from Costar (VWR International, Radnor, PA, USA), with 8.0 μm pore size PET track-etched membrane cell culture insert (BD Falcon, Franklin Lakes, NJ, USA)]. 0.5–1 × 10^6^ THP-1 cells were added to the upper chamber in 100 µL of serum-free medium (FBS-free adipocyte medium, AM-1-L1, ZenBio Inc.). The bottom well was filled with 600 µL cell-free supernatants from 3T3-L1-*in vitro* differentiated adipocytes or from *ex-vivo* isolated adipocytes, N-PEM, E0771 cells or their co-cultures in FBS-free AM-1-L1-SF medium or with the following recombinant paracrine factors and their antagonists: 10 and 50 ng/mL CCL2; 25 µg/mL CCL2 blocking antibody; 3, 100 and 500 ng/mL leptin; 100 nM LPrA2 (leptin receptor blocking peptide) and 2,5, 5, 10 and 100 µM lauric acid and 10 ng/mL Eritoran. After 2.5 h of incubation at 37 °C/5%CO_2_, the THP-1 cells that migrated to the bottom well were counted on the microscope. Assays were done in triplicate in migration chambers. 

### 2.7. ELISA

Cell and serum-free supernatants from *in vitro* differentiated 3T3-L1, *ex vivo* isolated adipocytes, N-PEM macrophages, E0771 mammary tumor cells and their co-cultures, or from N-PEMs macrophages pre-treated with paracrine factors, were harvested after 20 h incubation and tested by ELISA for the indicated cytokines/chemokines following manufacturers’ instructions. Murine leptin and CCL2 (MCP-1) were determined by ELISA kits from R&D Systems (Minneapolis, MN, USA), and murine IL-12p70 and IL-10 were analyzed using kits from eBiosciences (San Diego, CA, USA). 

### 2.8. Nitric Oxide

Nitric oxide was determined in cell supernatants from leptin-pretreated N-PEMs using the Griess colorimetric reaction as previously reported [17,25].

### 2.9. Free Fatty Acid (FFA) Determination

To measure the release of FFA from adipocytes, we analyzed adipocytes’ 48 h supernatants using an acyl-CoA oxidase-based enzymatic colorimetric methodology (WAKO Diagnostics, Richmond, VA, USA), following the manufacturer’s instructions and adapted for use on a Roche Cobas 6000 chemistry analyzer (Roche Diagnostics, Indianapolis, IN, USA). The intra- and inter- assay precision were <2.7% CV and 3.6%, respectively. 

### 2.10. Western Blot

N-PEMs (10^7^) were adhered to plastic tissue culture dishes and treated with 10, 20 and 100 ng/mL murine recombinant leptin with or without LPS (Sigma Aldrich, St. Louis, MO, USA) at 10 µg/mL for 20 h. Cells were then lysed in 100 µL RIPA lysis buffer and Western blots were performed as previously described [18,20]. Rabbit anti-mouse polyclonal antibodies (NFκBp50, NFκBp65), a goat anti-mouse polyclonal (Ob-R), a rabbit anti-mouse polyclonal (Notch 3) and a rabbit anti-mouse polyclonal (IRAK-1), all from Santa Cruz Biotechnologies (Santa Cruz, CA, USA), were used. Rabbit α-mouse STAT and phospho STAT (1 and 3) antibodies were from Cell Signaling (Cell Signaling Technology, Inc., Danvers, MA, USA). Rabbit α-mouse actin polyclonal antibody was obtained from Sigma-Aldrich and goat anti-rabbit IgG-HRP, bovine anti-goat IgG-HRP were both from Santa Cruz Biotechnologies. Densitometries were performed on the autoradiograms using Image J software (National Institutes of Health).

### 2.11. Luminex Assay

MILLIPLEX^R^ MAP Mouse Cytokine/Chemokine kit (Millipore, Billerica, MA, USA) was used for the simultaneous quantification of 32 mouse cytokines and chemokines in cell-free/serum-free culture supernatants from *in vitro* differentiated 3T3-L1, *ex-vivo* isolated adipocytes, N-PEM and E0771 cells and their co-cultures. Cytokines and chemokines analyzed were: Eotaxin, G-CSF, GM-CSF, IFNγ, IL-1α, IL-1β, IL-2, IL-3, IL-4, IL-5, IL-6, IL-7 IL-9, IL-10, IL-12p40, IL-12p70, IL-13, IL-15, IL-17, IP-10, KC, LIF, LIX, MCP-1, M-CSF, MIG, MIP-1α, MIP-1β, MIP-2, RANTES, TNFα and VEGF. The procedure was performed according to the manufacturer’s protocol. Plate reading was done on the Luminex 200 platform (Luminex Corp, Austin, TX, USA).

### 2.12. Flow Cytometry

N-PEMs were gently scraped from tissue culture plates, washed and counted after 20 h treatment with 10 ng/mL leptin. Fc receptors were blocked for 5 min with a Fc receptor antibody (eBioscience), and cells were stained with CD11b-FITC, CD115-PE, Gr-1-APC antibodies, all from eBioscience, and F4/80-PE Cy7 (BioLegend). Samples were acquired in a FACS Canto-II Analyzer and analyzed using FlowJo vX.0.6 (BD Biosciences, San Jose, CA, USA). Percentage of cells expressing the antibody and Mean Fluorescent Intensity (MFI) were determined.

### 2.13. Mice, Diets, Tumors and Treatments

C57BL6 female mice 6 weeks of age (NCI Frederick, MD) were used. Institutional animal care and use committee (IACUC) approved all the animal experiments. Diet-induced-obesity: To induce obesity, we used a high fat diet (HFD) containing 33%Kcal from fat (TD.03438, Harlan Laboratories, Inc., Madison, WI, USA), whereas a control low fat diet (LFD) containing 10%Kcal/fat was employed to generate lean control mice (TD.94048, Harlan Laboratories, Inc.). Five mice were kept per cage for both types of diets and body weight was evaluated weekly. Obese (and overweight) status was assessed by body weight (BW) increase: overweight was defined as a 15%–20% increase and obesity ≥25% BW higher than normal BW group (lean). Obesity-resistant mice were mice fed the 33% HFD where BW increased less than 15% weight. Tumor cell inoculation: Eight weeks after the diets were introduced, E0771 breast tumor cells, syngeneic to C57BL6 mice, were resuspended in PBS:Matrigel 1:1 and subcutaneously (s.c) injected in the fat pad of the fourth mammary gland in the lower abdomen at 2.5 × 10^5^ cells/50 µL/mouse. Primary tumor growth was measured at weekly intervals using calipers, and mice were kept in their respective diets until they were euthanized five weeks after tumor cell injection. Treatment with the leptin receptor antagonist peptide PEG-LPrA: A week after tumor cell inoculation, mice started treatments (50 uL/100 uM i.v. in tail vein) once a week with the peptide (PEG-LPrA2) or vehicle for 4 weeks, at the end of which they were euthanized. The peptide (PEG-LPrA2, pegylated leptin receptor antagonist peptide 2) was synthesized, purified, pegylated and provided by Dr. R.R Gonzalez-Perez. The mice were stratified into two major groups: (1) treated (PEG-LPrA2) and (2) treated with vehicle (designated either as “untreated” of “control” in Figure 9). Within each major group, there were 4 subgroups: lean, obese-resistant, overweight and obese, each with *n* = 10 per subgroup. Right before euthanasia, serum samples were obtained and frozen at −80 °C for leptin, CCL2, glucose, and insulin determinations. Tumor detection: Mammary glands were palpated weekly for tumor formation. Tumor growth was determined every week in live animals by caliper measurement (as an estimated measure of growth, ╥/6 × width^2^ × length, where the width is the smaller of the two measurements) and upon euthanasia. Tumors were fixed in 10% formaldehyde for further histological and immunohistochemistry studies.

### 2.14. Histology and Immunohistochemistry

Tissue sections were stained with Hematoxylin and Eosin (H&E) to reveal the histology of the tumors. Immunohistochemistry (IHC) was performed as previously reported [20] using the VECTASTAIN ABC Kit as described by the manufacturer (Vectors Laboratories, Burlingame, CA, USA). The following were the primary antibodies used for IHC: rat monoclonal anti-mouse F4/80 (Abcam, Cambridge, MA, USA), rat monoclonal anti-mouse IL-10 (Santa Cruz), rat monoclonal anti-mouse IL-12 (Santa Cruz), and rabbit polyclonal anti-Ob-R (H300, Santa Cruz, which recognizes the long and short forms of the leptin receptor). Antigen retrieval was performed using the following conditions: for F4/80, IL-10 and IL-12 it was made in Citrate Buffer (Antigen Unmasking Solution, Vector Laboratories), whereas for leptin receptor (Ob-R), it was carried out in High pH Buffer (Antigen Unmasking Solution High pH, Vector Laboratories). First antibodies were incubated overnight at 4 °C. 

### 2.15. Determination of Glucose, Leptin, CCL2 and Insulin in Sera

The serum levels of glucose (Colorimetric Assay Kit, Cayman Chemical Company, Ann Arbor, MI, USA), insulin (Mouse Insulin ELISA, Mercodia, Uppsala, Sweden), leptin and CCL2 (both by ELISAS from R & D System) were determined following the manufacturer’s instructions.

### 2.16. Statistical Analysis

Prism software was used to statistically analyze our results. One or two-way ANOVAs were used when comparing more than two experimental groups; a significant ANOVA test shows that the different groups are statistically different among themselves as a group. However, to compare pairs of groups, ANOVA was then followed by Tukey’s Multiple Comparisons Test (TMCT) or by Sidak’s Multiple Comparison Test (SMCT), which allow comparing the means between pairs of groups among the larger group analyzed by ANOVA. Levels of significance were provided by Prism analysis (*p* ≤ 0.05 *, *p* ≤ 0.01 **, *p* ≤ 0.001 *** and *p* ≤ 0.0001 ****. These *p* values apply to all presented figures using the different statistical tests. The Multiple Comparisons derived from significant ANOVAs, Tukey’s or Sidak’s (whether significant or not) are shown for each figure in Supplemental Data, and only the significant values of biological relevance are depicted in each figure. Paired Student’s *t*-test was used when means between two groups were initially compared to analyze their statistical difference. Error bars represent standard error of the mean (SEM) and alpha was set at 5%. 

## 3. Results

### 3.1. Lipolysis in Adipocytes Is Modulated by Tumor Cells and Macrophages

**Figure 1:**
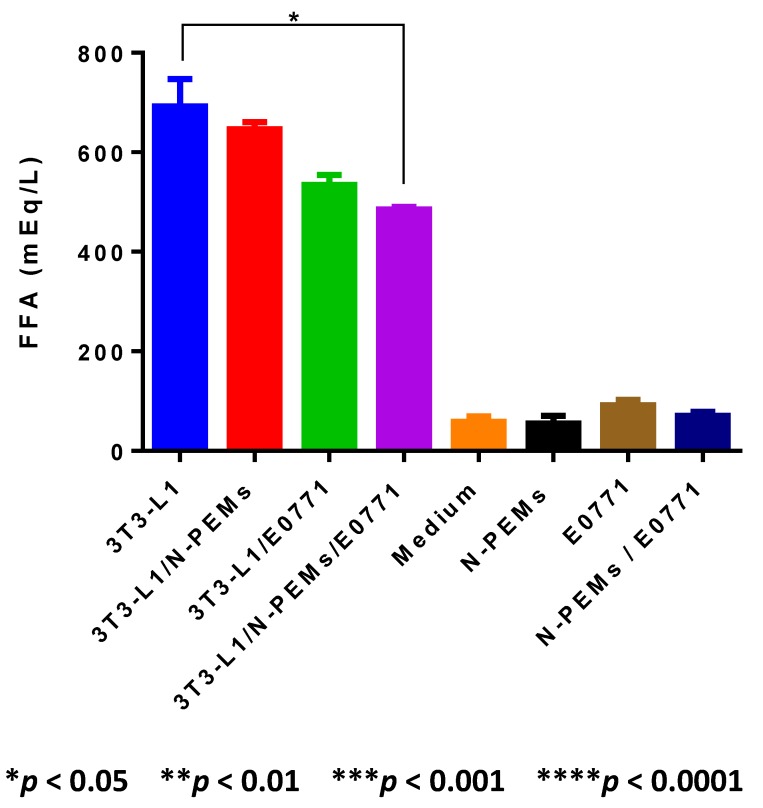
Free fatty acid (FFA) concentration in mammary tumors may be modulated by cells of the mammary tumor microenvironment. Co-culture of adipocytes, macrophages and mammary tumor cells results in reduced amounts of FFA detected in the co-culture cell supernatant, as compared with the amounts released by adipocytes only through lipolysis, as determined by acyl-CoA oxidase-based enzymatic colorimetric methodology in 48 h supernatants from 3T3-L1 murine adipocytes *in vitro* differentiated for 11 days and co-cultured with macrophages, with mammary tumor cells or with both. Data are representative of one of three independent experiments showing similar results; each sample was tested in duplicates.

To determine how lipolysis (the release of glycerol and free fatty acids, FFA, from triglycerides) within adipocytes in the breast gland could be modulated by the breast tumor microenvironment, we measured the release of FFA by adipocytes in the presence of mammary tumor cells and macrophages. To this end, *in vitro* differentiated 3T3-L1 murine adipocytes were co-cultured with mouse mammary tumor cells (E0771) and/or murine peritoneal elicited macrophages (N-PEMs). Our data demonstrate (Figure 1) that co-culturing adipocytes with either macrophages or tumor cells resulted in less FFA detected in the cell co-culture supernatant, with a greater downregulation exerted when adipocytes were co-cultured with tumor cells than with macrophages, and the strongest decrease detected upon co-culture of adipocytes with both other cell types, a setting that mimics the breast tumor microenvironment. Our results also show that mammary tumor cells, macrophages or their co-culture do not produce significant amounts of FFA. All groups were significantly different among themselves as assessed by one-way ANOVA (* *p* < 0.05), and statistical significance of the comparison between 3T3-L1 adipocytes and their co-culture with N-PEMs and E0771 cells is depicted in Figure 1 using Tukey’s Multiple Comparison Test (TMCT from now on); the complete statistical analysis for all comparisons in the figure is shown in Supplementary Data.

### 3.2. In Vitro Differentiated and in vivo Isolated Adipocytes Are very Different in Their Leptin Production and in Their Sensitivity to Stimulation by Macrophages and Tumor Cells

**Figure 2:**
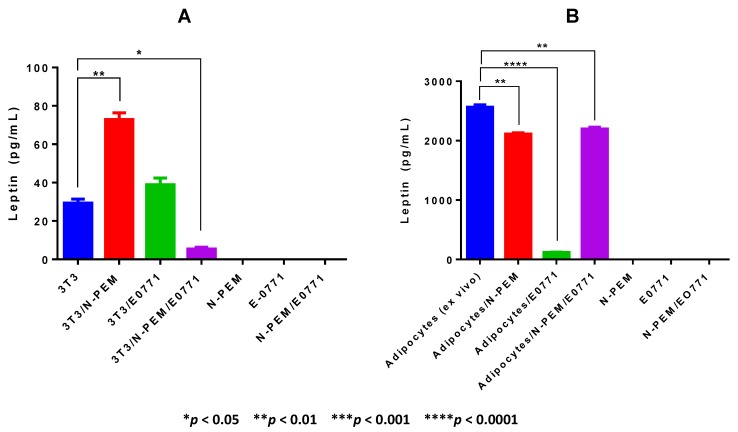
*In vitro* differentiated adipocytes significantly differ from adipocytes *ex vivo* isolated from visceral obese adipose tissue in their leptin secretion: leptin is a major marker of obesity and not of adipocyte differentiation. 48 h supernatants from *in vitro* differentiated (**A**) and *ex vivo* isolated adipocytes, (**B**) co-cultured with macrophages and tumor cells were analyzed for leptin production using ELISA. Data are representative of one of three independent experiments showing similar results; each sample was tested in duplicates. One-way ANOVA analysis showed differences among all the samples in both figures (**A**: *** *p* < 0.001 and **B**: **** *p* < 0.0001); TMCT comparisons are depicted in the figures.

To mimic the production of leptin by adipocytes in the mammary tumor microenvironment, adipocytes from two different sources (*in vitro* differentiated and *ex vivo* isolated from obese visceral adipose tissue) were co-cultured with macrophages and tumor cells. Figure 2A presents the results of leptin production by *in vitro* differentiated 3T3-L1 adipocytes. Co-culturing 3T3-L1 adipocytes with macrophages substantially enhances leptin production by adipocytes, even though macrophages alone do not produce leptin. E0771 tumor cells, contrary to other mammary/breast tumor cells, do not produce leptin, and co-culturing 3T3-L1 adipocytes with E0771 cells does not significantly contribute to an increase in leptin production by these adipocytes. Interestingly, the co-culture of the three cell types results in a profound downregulation of leptin production. Figure 2B shows the results of leptin production by adipocytes *ex vivo* isolated from visceral fat of obese female mice. In contrast to the data obtained from *in vitro* differentiated adipocytes (Figure 2A), *ex vivo* isolated adipocytes from obese mice are the major source of leptin production. Further, as opposed to *in vitro* differentiated 3T3-L1 cells, obese adipocytes are not additionally stimulated to produce leptin by co-culture with either macrophages or tumor cells. On the contrary, isolated obese adipocytes cultured with N-PEMs, but especially with E0771, significantly downregulate leptin production, and the co-culture of the three cell types resulted in a significant decrease in leptin production, as also occurred with 3T3-L1 adipocytes. Importantly, the levels of leptin production by *ex vivo* isolated adipocytes from obese mice are over 80-fold higher than the ones produced by 3T3-L1 *in vitro* differentiated adipocytes. These results suggest that leptin is more a marker of obesity than of differentiation, since adipocytes from obese adipose tissue but not differentiated 3T3-L1 adipocytes are the major producers of leptin. 

### 3.3. Cross Talk between Adipocytes from Obese Mice, Tumor Cells and Macrophages Results in the Highest Production of CCL2

**Figure 3:**
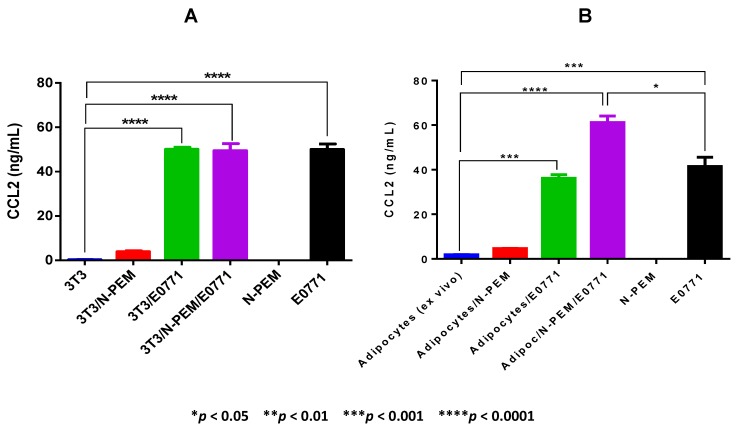
Co-culture of adipocytes, tumor cells and macrophages reveals the highest production of CCL2 (MCP-1). 48 h supernatants from *in vitro* differentiated (**A**) and *ex vivo* isolated (**B**) adipocytes co-cultured with macrophages and tumor cells were analyzed for CCL2 production using ELISA. Data are representative of one of three independent experiments showing similar results; each sample was tested in duplicates. One-way ANOVA analysis showed differences among all the samples in both figures (**** *p* < 0.0001), and TMCT comparisons are depicted in the figures.

The CCL2 chemokine (MCP-1) is a major chemoattractant of macrophages to tissues. We analyzed its production by the three main cell types under investigation to determine their respective contribution to this chemokine’s production in the mammary tumor microenvironment. Our results (Figure 3) show that among the three cell types studied individually, E0771 mammary tumor cells are by far the main producers of CCL2, compared to macrophages and adipocytes. 

Interestingly, the co-culture of the three cell types resulted the highest expression of CCL2 when adipocytes *ex vivo* isolated from obese adipose tissue were used (Figure 3B), more than when *in vitro* differentiated adipocytes were used (Figure 3A). Co-culturing *in vitro* differentiated adipocytes with tumor cells or with tumor cells and macrophages resulted in similar levels of CCL2 production to the ones expressed by E0771 cells (Figure 3A). We show that macrophages do not produce detectable levels of CCL2, yet *ex vivo* isolated adipocytes from obese adipose tissue (Figure 3B) produce higher amounts than *in vitro* differentiated 3T3-L1 cells (Figure 3A), although the levels of adipocyte CCL2 production are in general very low when compared to the levels produced by tumor cells. Despite the fact that adipocytes and macrophages by themselves produce almost no CCL2, both *in vitro* differentiated and *in vivo* isolated adipocytes co-cultured with macrophages result in the detection of measurable amounts of CCL2 in the supernatants (ns compared with adipocytes only in each case, 3A and 3B, TMCT). Importantly, in the absence of tumor cells, the production of CCL2 is negligible (3A and 3B). Taken together our results demonstrate that it is the crosstalk between adipocytes from obese mice, macrophages and tumor cells—which is the setting that more closely resembles the mammary tumor microenvironment of obese females—the condition that results in the generation of the highest amount of CCL2, the main macrophage chemoattractant to tissues. 

### 3.4. The Combined Action of Adipocytes, Tumor Cells and Macrophages, as well as the Combined Effect of Leptin, Lauric Acid and CCL2 Enhances Macrophage Chemotaxis

**Figure 4:**
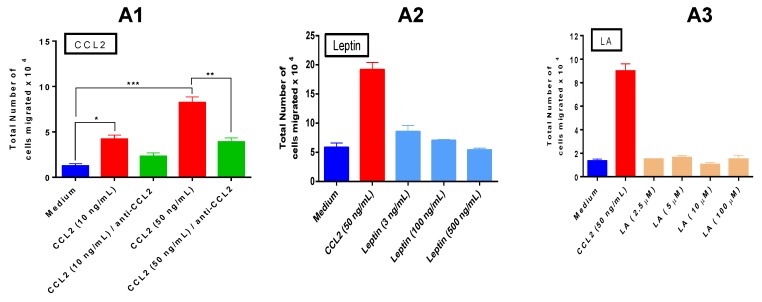

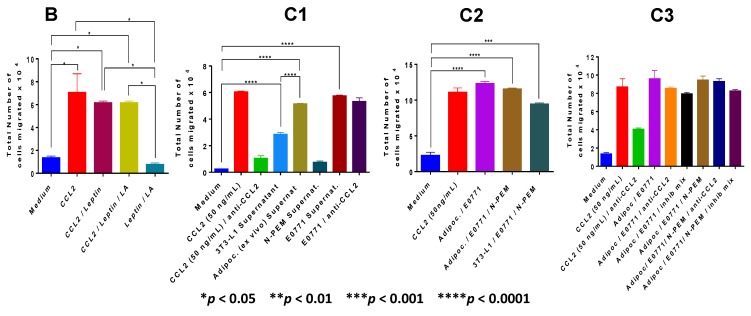
Migration studies with human monocytic cell line THP1. Chemotaxis experiments were conducted for 2.5 h at 37 °C. Top Chamber (ThinCert 8 µm pores): 1 × 10^6^ THP1cells in 100 µL vol. Bottom chamber contained the chemoattractant cells supernatants or recombinant/synthetic factors in 600 µL volume. (**A1**) CCL2 is a major monocyte chemoattractant, anti-CCL2 antibody (25 µg/mL) significantly reduces its activity. (**A2**) Increasing leptin concentrations has weak and ns chemotactic activity. (**A3**) Lauric acid has no chemoattractive properties on monocyte. (**B**) Effect of the different paracrine factors: The combination of CCL2, leptin and lauric acid does not significantly modify the monocyte chemoattraction comparable to the one exerted by CCL2, but in absence of CCL2 there is no chemotaxis. (**C**) Effects of 48 h supernatants from the different cells of interest (adipocytes, macrophages and tumor cells) in monocyte recruitment, without and with the paracrine factors inhibitors. CCL2 was used at 50 ng/mL, leptin at 10 ng/mL and LA at 5 µM in **B** and **C**. Anti-CCL2 blocking antibody was used at 25 µg/mL, leptin receptor inhibitor LPrA2 was used at 100 nM and TLR4 inhibitor Eritoran was used at 10 ng/mL. Data are representative of one of three independent experiments showing similar results; each sample was tested in triplicates. One-way ANOVA analysis showed significant differences among all the samples in the different figures: **A1**, **A2**: *** *p* < 0.001; **A3**: **** *p* < 0.0001; **B**: ** *p* < 0.01, and, **C1**, **C2** and **C3** **** *p* < 0.0001; TCMT comparisons are depicted in the figures.

Blood monocytes are recruited to tissues, where they differentiate into macrophages. We analyzed the induction of chemotaxis on THP1 human monocytes by supernatants of the three main cell types and their co-cultures, and we also used recombinant and/or synthetic forms of some of the paracrine factors we propose may be present in the breast tumor microenvironment of obese females, e.g., leptin, the saturated FFA lauric acid and CCL2. We showed (Figure 3) that a main macrophage chemoattractant, chemokine CCL2, is produced in elevated amounts by the E0771 mammary tumor cells, which is enhanced by the crosstalk between adipocytes from obese adipose tissues with E0771 cells and macrophages (Figure 3B). We thus used CCL2 as the gold standard/positive control in our monocyte chemoattraction/migration studies (Figure 4).

Figure 4A1 presents our results using a migration or transwell assay to study THP1 monocytes chemotaxis, showing the potent chemoattractive effects of CCL2 on these cells in a dose-response fashion, and how an anti-CCL2 blocking antibody significantly reduces monocyte chemotaxis by CCL2. Figure 4A2 shows a modest monocyte chemoattractive effect of the lowest concentration of the adipokine leptin used (ns compared to media, TMCT), and how migration decreases even further although not significantly with higher leptin concentrations, compared to the strong effects of CCL2. Among the FFAs, saturated fatty acids exhibit the highest inflammatory properties [26]. Lauric acid (LA) is a saturated 12-carbon atom fatty acid that has been reported as a ligand of TLR4 [27], a TLR highly expressed by macrophages. We examined whether increasing concentrations of lauric acid could attract monocytes. Figure 4A3 shows the absence of monocyte chemotactic properties of this fatty acid (ns compared to media, TMCT). Interestingly however, the mixture of the three paracrine factors (CCL2, leptin and lauric acid) elicited a significant chemoattraction of monocytes, as high as the one exerted by CCL2 (ns compared to CCL2, TMCT), although in the absence of CCL2 there was no migration (Figure 4B). We then examined the effects of supernatants from the different cells of interest (adipocytes, macrophages and tumor cells) in monocyte recruitment after 48 h in culture (Figure 4C). Figure 4C1 reveals that supernatants from adipocytes (*in vitro* differentiated or *ex vivo* isolated) acting directly on monocytes exert strong chemotaxis, with *ex vivo* isolated adipocytes from obese adipose tissue exerting a much stronger chemotaxis than 3T3-L1 adipocytes. Interestingly, supernatants from macrophages exert a negligible chemotaxis on monocytes (Figure 4C1). On the other hand, supernatants from E0771 tumor cells, which are the major producer of CCL2 among the three cell types studies, exerted the strongest chemotaxis among the individual cellular supernatants analyzed, comparable to CCL2 (Figure 4C1). When analyzing the effects of the different cell co-cultures on chemotaxis, interestingly, supernatants from the three cell types co-cultured together induce higher chemotaxis when *ex vivo* isolated adipocytes from obese adipose tissue were used than when 3T3-L1 adipocytes were present (Figure 4C2). The co-culture of *ex vivo* isolated adipocytes from obese adipose tissue with tumor cells—without macrophages—exhibits a chemotaxis comparable to the one induced by CCL2 by itself and to the one induced by the co-culture of the *ex vivo* adipocytes + E0771 + macrophages (Figure 4C2). Interestingly, the strong chemotactic effect of E0771 cells could not be abrogated with anti-CCL2 blocking antibody (Figure 4C1), suggesting the existence of additional chemotactic factors produced by E0771 or of very high amounts of CCL2 produced by E0771 cells as previously showed in Figure 3, which might probably require a higher amount of the anti CCL2 blocking antibody. To distinguish between these possibilities, in addition to the blocking antibody for CCL2, we used specific inhibitors for leptin receptor (LPrA2) and for TLR4 (E5564, Eritoran). We thus added CCL2 blocking antibody as well as the mixture of the three inhibitors (inhibitor mix: anti CCL2 antibody + anti leptin receptor peptide + Eritoran) to different cell co-culture supernatants. Figure 4C3 demonstrates that using the inhibitor mix on the *ex vivo* adipocyte + E0771 co-culture supernatant or on the *ex vivo* adipocyte + E0771 + macrophages supernatant resulted in a nonsignificant decrease on chemotaxis (ns in both cases when the conditions without and with the mix were compared, TMCT), suggesting the existence of other paracrine factors, in addition to the examined ones, produced by the crosstalk between those different cell types that may account for monocyte chemotaxis to the tumor microenvironment. All in all our data indicate that the interaction of tumor cells and adipocytes—particularly those from obese adipose tissue—with macrophages, plays an important role in the recruitment of blood monocytes to the mammary tumor microenvironment. Also, that in addition to CCL2, leptin and lauric acid, there are additional unidentified paracrine factors promoting monocyte chemotaxis to the obese mammary tumor microenvironment.

### 3.5. Identification of Additional Cytokines, Chemokines and Growth Factors in the Supernatants of Adipocytes, Tumor Cells, Macrophages and Their Co-Cultures

**Figure 5:**
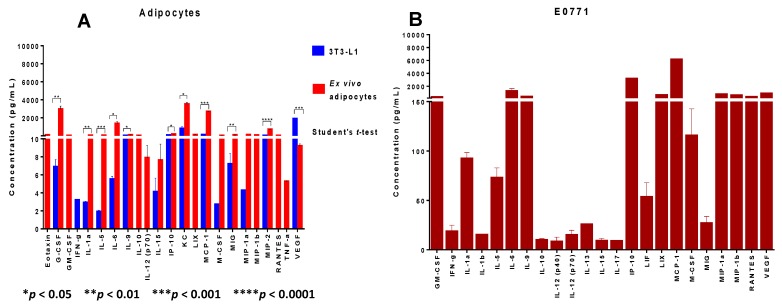

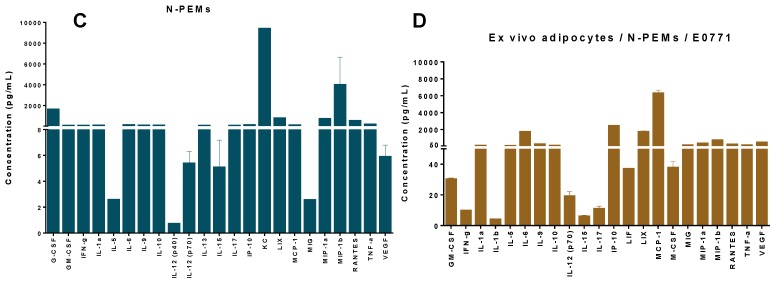
Cytokines, chemokines and growth factors detected in cell supernatants from adipocytes, mammary tumor cells, macrophages and their co-cultures. 48 h supernatants from the three different cell types and their co-cultures were analyzed for cytokines, chemokines and growth factors using Luminex analysis. (**A**) Comparison of the proteins secreted by *in vitro* differentiated 3T3-L1 adipocytes and *ex vivo* isolated adipocytes, showing statistical differences (Student’s *t-*test) between the two types of adipocytes compared to each other. (**B**) Cytokines/chemokines/chemokines/growth factors detected in E0771 cell supernatants. (**C**) Cytokines/chemokines/growth factors secreted by peritoneal elicited macrophages. (**D**) Cytokines/chemokines/growth factors secreted by co-cultures between *ex-vivo* isolated adipocytes, macrophages and E0771 mammary tumor cells. Data are representative of one of two independent experiments showing similar results; each sample was tested in duplicates.

To investigate if additional chemotactic molecules are secreted by adipocytes, tumor cells and macrophages individually, as well as in their co-culture, we performed Multiplex (Luminex) studies. Figure 5A shows our results when comparing 48 h supernatants from *in vitro* differentiated adipocytes with 48 h supernatants from adipocytes *ex vivo* isolated from obese fat tissue. In general molecules were expressed at much higher levels by *ex vivo* isolated adipocytes from obese mice than by *in vitro* differentiated adipocytes. IFNγ was only produced by *in vitro* differentiated 3T3-L1 adipocytes, whereas eotaxin, GM-CSF, IL-10, IL-12p70, LIX, MIP-1β (CCL4), RANTES (CCL5) and TNFα were found only in supernatants from adipocytes *ex vivo* isolated from obese adipose tissue. There were statistically significant differences between the expression levels of several of the molecules produced by these two types of adipocytes: unpaired Student’s *t*-test analysis showed significantly higher levels of IL-6, IL-9, IP-10 (CXCL10), KC (CXCL1), G-CSF, MIG (CXCL9), IL-1α, MCP-1, VEGF, IL-5, and MIP-2 (CXCL2) produced by *ex vivo* isolated adipocytes compared to the levels expressed by 3T3-L1* in vitro* differentiated adipocytes, suggesting greater capacity of *ex vivo* adipocytes from obese fat tissue to recruit and activate monocytes, neutrophils and polymorphonuclear leukocytes as well as higher inflammatory and angiogenic effects. Figure 5B shows Luminex analysis of E0771 tumor cells supernatants (48 h), which confirm very high expression of CCL2 (MCP-1) and also of IP-10 (CXCL10) by these cells, as well as of IL-6 and VEGF. Not detected in E0771 supernatants were Eotaxin, IL-2, IL-3, IL-4, IL-7, MIP-2 (CXCL2) and TNF-α, and above the range determined by the Luminex’s standard curve were G-CSF (>30,787 pg/mL) and KC (CXCL1) (>25,314 pg/mL), both values approximately extrapolated from the curve. Peritoneal elicited macrophages (Figure 5C) constitutively produce very large amounts of CXCL1 (KC) and MIP-1β (CCL4) as well as large amounts of G-CSF, but express no detectable levels of Eotaxin, IL-1β, IL-2, IL-3, IL-4, IL-7, LIF and M-CSF, and above the range levels MIP-2 (CXCL2, >13,109 pg/mL). Interestingly, co-culture of the three cell types (Figure 5D) revealed very high levels of CCL2 and IP-10, both very similar to the amounts found in E0771 supernatants, and also high levels of IL-6 and LIX, an inductor of CCL2, as well as high levels of MIP-1β (CCL4) and VEGF. Furthermore, these co-cultures showed non-detectable levels of Eotaxin, IL-2, IL-3, IL-4, IL-7, IL-12 (p40), IL-13, and exhibited above the range values of G-CSF (>30,787 pg/mL), KC (CXCL1) (>25,314 pg/mL), MIP-2 (CXCL2) (>13,109). Considered together, our data demonstrate that adipocytes from obese adipose tissue, macrophages and mammary tumor cells, by themselves or interacting together, express numerous cytokines, chemokines and growth factors with chemotactic, pro-inflammatory, pro-angiogenic and tumor-promoting activities.

### 3.6. Leptin Modulates Cytokine, Chemokine and Growth Factor Production in Macrophages

**Figure 6:**
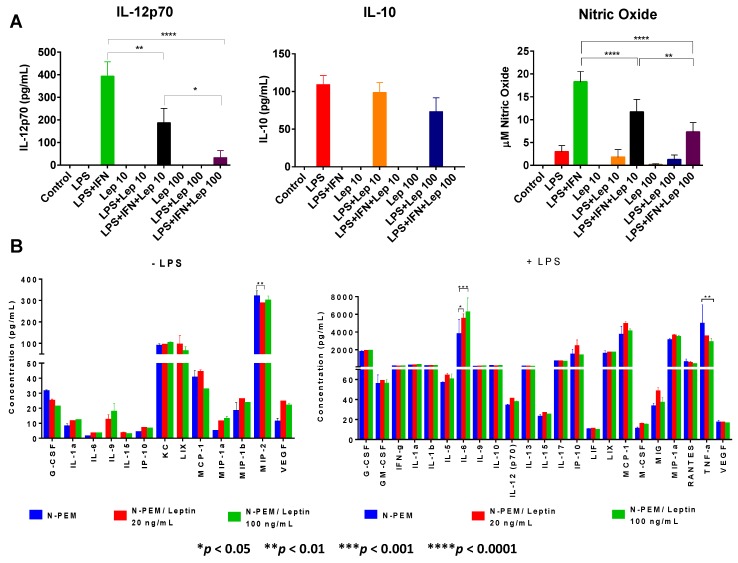
Leptin regulates expression of cytokines and chemokines in macrophages. Peritoneal elicited macrophages were pre-treated with increasing concentrations of leptin (0, 10 and 100 ng/mL) and activated with LPS (10 µg/mL) or with and LPS+IFNγ (IFNγ at 100 u/mL) for 20 h, and supernatants were collected and tested for IL-12p70 and IL-10 expression by ELISA and for nitric oxide (NO) production via the Griess reaction (**A**). In other experiments, constitutive and LPS-activated macrophages (**B**) were treated with increasing concentrations of leptin (0, 20 and 100 ng/mL) for 20 h and their supernatants examined by Luminex. Data are representative of one of three independent experiments in each case, which yield similar results, where each sample was tested in duplicates. One-way ANOVA analysis showed significant differences among all the samples in the figures: in **A** *** *p* < 0.0001 and in **B** ** *p* < 0.0001; TCMT comparisons are depicted in the figures.

Given the recognized tumor-promoting role of leptin in breast cancer [28,29], we analyzed whether in addition to its possible chemotactic function in recruiting macrophages to the tumor microenvironment, leptin could also regulate macrophage M1/M2 profiles in the mammary tumor microenvironment, particularly in obesity. To this aim, we studied macrophage expression of central pro- and anti-inflammatory cytokines, chemokines and growth factors upon pre-treatment of peritoneal elicited macrophages (N-PEMs) with increasing leptin concentrations activated or not with LPS and IFNγ. Our results (Figure 6A) demonstrate that N-PEMs, which express the pro-inflammatory/anti-tumor cytokine IL-12p70 only after stimulation with LPS and IFNγ, profoundly downregulate IL-12p70 expression (M1 marker) when exposed to increasing concentrations of leptin (0, 10 and 100 ng/mL). A similar result was observed for nitric oxide (NO), a pro-inflammatory reactive nitrogen intermediate (RNI) and free radical, crucially involved in the killing activities of M1 macrophages. In contrast to IL-12p70, NO is produced by N-PEMs upon LPS activation only, and is further upregulated upon macrophage activation with LPS+IFNγ. We found that leptin decreases macrophage production of NO after LPS+IFNγ activation (Figure 6A), and that upon LPS activation only, there is a nonsignificant tendency for such downregulation to occur. As opposed to IL-12p70, the anti-inflammatory cytokine IL-10 (M2 marker) is produced by N-PEMs after LPS, but not upon LPS+IFNγ stimulation. Interestingly though, no significant changes in the production of IL-10 were observed in macrophages treated with leptin (Figure 6A). To examine the possibility that a wider range of pro- and anti-inflammatory macrophage cytokines, chemokines and growth factors could be modulated by leptin, we performed Luminex analysis in 20-hour supernatants from constitutive and LPS-activated macrophages pre-treated with 20 and 100 ng/mL leptin (Figure 6B). Our results show that 20 ng/mL leptin significantly downregulates the constitutive expression of the pro-inflammatory chemokine CXCL2 (MIP-2), chemotactic for monocytes (Figure 6B). We report that in LPS-activated macrophages, leptin alters in opposite ways two different pro-inflammatory cytokines: it significantly upregulates IL-6 expression but dowregulates TNFα in a dose-response fashion (Figure 6B). Taken together, our results reveal that leptin decreases the expression of critical pro-inflammatory cytokines, chemokines and RNI in macrophages [IL-12, TNFα, MIP-2 (CXCL2) and NO] yet significantly upregulates the pro-inflammatory and tumor-promoting IL-6, although it does not seem to modulate main anti-inflammatory molecules in macrophages (as IL-10).

### 3.7. Leptin Decreases the Expression of Myeloid Differentiation Markers in Macrophages

**Figure 7:**
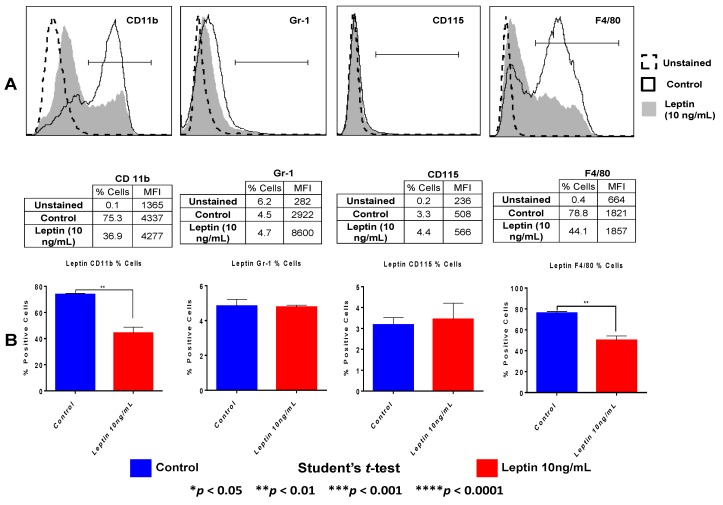
Leptin impairs expression of myeloid differentiation markers in macrophages. (**A**) Flow cytometric analysis of CD11b, F4/80, CD115 and Gr-1 expression in resting N-PEMs pre- treated with leptin at 10 ng/mL for 20 h shows that macrophages exposed to leptin downregulate the percentage of cells that express F4/80 and CD11b. Unstained macrophages were compared with untreated (control) and leptin-treated macrophages stained with the antibodies for those markers. Flow cytometry results were analyzed using Flow Jo software; percentages (%) of cells expressing the respective markers as well as the mean fluorescence intensity value (MFI) of each marker were analyzed. The figure presents the results of a representative experiment with macrophages from an individual mouse; three different experiments were made with five mice per experiment; (**B**) Histograms showing the statistical analysis made using Student’s *t*-test to compare the means of the percentages of cells expressing the analyzed markers in macrophages from *n* = 15 mice used in the three experiments.

Macrophages normally express several myeloid markers, and the elevated expression of some of them is considered indicative of macrophage differentiation [18]. We previously demonstrated that peritoneal and tumor-associated macrophages from mammary tumor-bearing mice exhibit decreased mean fluorescent expression (MFI) of myeloid differentiation markers F4/80, CD11b, CD68 and CD115 (MCSF receptor) yet upregulate Gr-1 [18,20]. We were now interested in investigating whether exposure to leptin may modulate the expression of these markers, and thus, could regulate differentiation in macrophages. Our results (Figure 7) demonstrate that leptin treatment decreases the percentage of cells expressing F4/80 and CD11b (% positive cells) in macrophages but it does not significantly impact Gr-1 or CD115 expression.

### 3.8. Leptin Modulates the Expression of Central Pro-Inflammatory Transcription Factors, Membrane Signaling Receptors and of its own Receptor in Macrophages

Leptin produced by adipocytes in the mammary/breast tumor microenvironment of obese females may not only act on tumor cells to make them acquire a more aggressive phenotype [30,31], but as we showed here in the *in vitro* experiments, acting together with other paracrine factors from the tumor microenvironment may also contribute to increasing macrophage recruitment to the tumor. Leptin also regulates macrophage pro-inflammatory functions, phenotypes and differentiation. It could furthermore act on macrophages in the tumor microenvironment (TAMs), or on mammary tumor-distal (peritoneal) macrophages, to modulate other tumor-promoting activities. We have previously shown that TAMs overexpress NFκBp50 homodimers as well as STAT1/pSTAT1 and STAT3/pSTAT3 transcription factors [20]. We have also revealed that peritoneal macrophages from tumor bearers display different expression patterns of these same transcription factors compared to the ones seen in TAMs [18].

**Figure 8:**
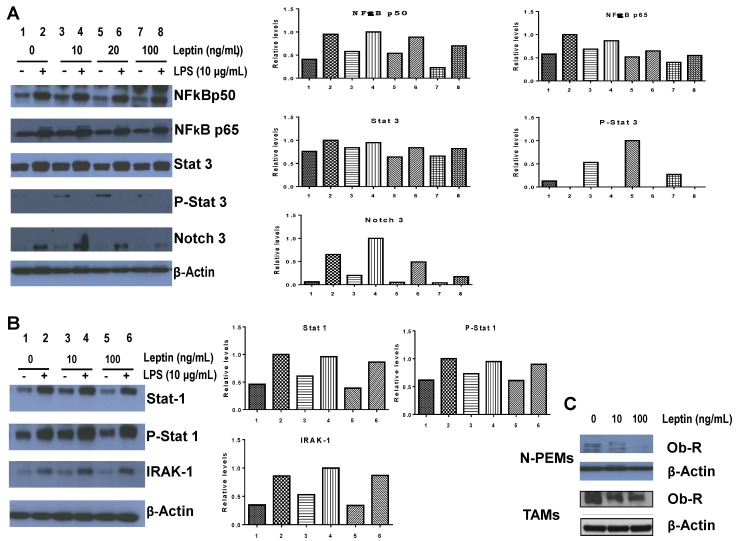
Leptin regulates the expression of pro-inflammatory/tumor-promoting transcription factors, membrane receptors and signaling intermediates, as well as of its own receptor in macrophages. N-PEMs (and also TAMs in C) were pre-treated with increasing concentrations of leptin for 20 h with or without LPS, whole cell protein was isolated and Western blots were performed. Densitometries of the autoradiograms are shown.

Figure 8A,B illustrate the effects of pre-treating N-PEMs with increasing concentrations of leptin on their expression of central pro-inflammatory/tumor-promoting transcription factors (NFκB, STAT1/3) and membrane signaling receptors or signaling intermediates (Notch 3, IRAK-1). Our results confirm that leptin downregulates constitutive NFκB p50 expression and constitutive and LPS-induced expression of NFκBp65 but upregulates LPS-induced p-STAT3 expression. We report that at low concentrations, leptin increases Notch 3 expression but that at higher concentrations it has a downregulating effect in Notch 3 (Figure 8A), as we observed was the case with chemotaxis (Figure 4A2). Interestingly, leptin does not modulate expression of STAT-1/pSTAT1 but upregulates IRAK-1 in macrophages (Figure 8B). We also examined whether the presence of leptin might control the expression of its own receptor in macrophages. Figure 8C shows that pre-treating peritoneal N-PEMs as well as TAMs with increasing concentrations of leptin profoundly downregulates both long (Ob-Rb) and short (Ob-Ra) forms of leptin’s receptor in both types of macrophages. 

Taken together, our *in vitro* results modeling the role of leptin in the mammary tumor microenvironment of obese females suggest that leptin’s presence where it is produced by adipocytes, the bAT, seems to play a moderate role by itself in macrophage recruitment. However it does play a significant role in regulating macrophage phenotypes, activation and function. These pleiotropic effects of leptin on macrophages exist despite the fact that leptin induces downregulation of its own receptor in these cells, as we showed for both peripheral and tumor-associated macrophages. Importantly, we have demonstrated that the crosstalk of macrophages with tumor cells and adipocytes results in a powerful interaction between many different paracrine factors whose integrated action is one that promotes macrophage recruitment and tumor development.

### 3.9. Obesity—but not Leptin Alone—Plays a Role in Tumor Progression in the E0771 Mouse Mammary Tumor Model Fed 33% HFD

**Figure 9:**
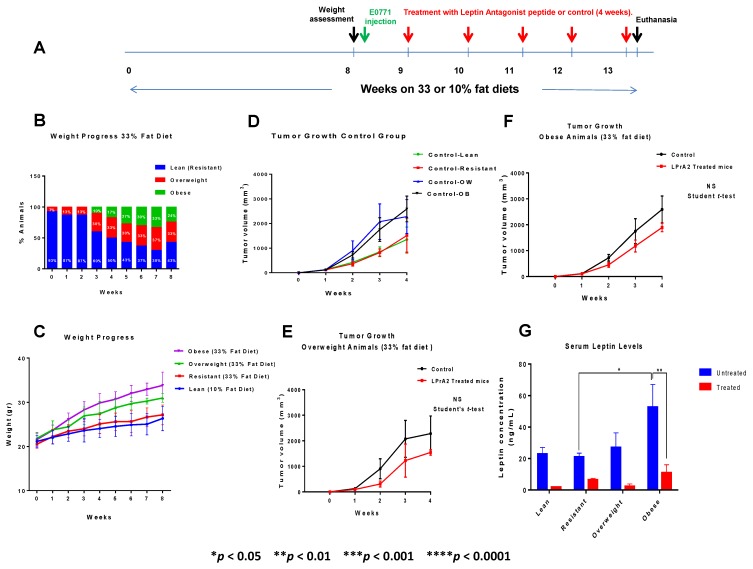
*In vivo* experiments: weight control and tumor progression. (**A**) Experimental design of the *in vivo* experiments. (**B**) Feeding 33%Kcal/fat HFD results in a rapid splitting of the mice weights into obese, overweight and obese resistant animals in approximately 7–8 weeks after the start of the diets. (**C**) Weight progress in all the mice fed both HF and LF diets during the first eight weeks before tumor cells were implanted. (**D**) General effect of body weight on tumor growth. (**E**) Effect of treatment with leptin antagonist peptide on tumor growth in overweight mice. (**F**) Effect of treatment with leptin antagonist peptide on tumor growth in obese mice. (**G**) Association between body weight and systemic (serum) levels of leptin in tumor bearers with different body weights untreated or treated with the leptin inhibitor. Each experimental subgroup was comprised of ten (10) mice.

To validate our *in vitro* studies where we modeled the mammary tumor microenvironment in obese mice, we carried out *in vivo* experiments using the E0771 mouse mammary tumor model syngeneic to the C57BL6 strain with diet-induced obese (DIO) and lean female mice. We investigated whether body weight increase and modulation of leptin signaling might affect macrophage recruitment/activation and tumor progression. Figure 9 presents the main results of our *in vivo* experiments in which some mice were fed a 33% High fat diet (HFD) and others were given a 10% low fat diet (LFD) as control. Figure 9A shows the experimental design that was followed, as explained in the Experimental Section.

Weight Progress: The 33% HFD closely represents the human Western diet much better than other experimental HFDs, because as in human populations, consumption of this diet results in a certain percentage of mice becoming obese, others becoming overweight, and even others remaining resistant to the diet. After only 6–7 weeks on this diet, obesity was developed in approximately 30% of the mice, overweight was seen in about 30% of the mice, and roughly 30% of the mice showed body weight (BW) lower than BW+2SD of the normal BW control group so were deemed obesity-resistant (Figure 9B), as previously described [32]. In earlier results with the 33% HFD, when mice were individually kept in cages, it took several months for the animals to split up into the three groups (obese, overweight and obese-resistant) [33]. In contrast to these results, we showed (Figure 9B) that when mice were kept in groups of five per cage, as we did here, they reached their respective weight category much sooner, suggesting they were eating more or were absorbing/metabolizing better the food they ate when they were living in groups. After tumor cells were injected and tumors started developing, all mice started losing weight progressively, regardless of their anti-leptin signaling treatment or lack thereof, and they were euthanized four weeks after starting treatments.

We assessed the kinetics of weight increase in all the mice fed low and high fat diets during the first 8 weeks of feeding, before they were injected with the tumor cells (Figure 9C). Interestingly, mice separated into the different weight categories (lean (low-fat), obesity-resistant, overweight and obese) in a statistically significant fashion as early as 2 weeks after they started on the diets [(** *p* < 0.01, comparison of lean (10% LFD) *vs*. obese (33% HFD) mice and * *p* < 0.05 comparison of obesity-resistant (33% HFD) *vs*. obese (33% HFD), TMCT)]. 

In weeks 3 and 4 and through the end of the pre-tumor period (week 8), the differences in weight among the various groups remained: lean *vs*. obese was highly significant, **** *p* < 0.0001, whereas obesity-resistant *vs*. obese was also highly significant (**** *p* < 0.0001, TMCT), and no significant differences between lean and obesity-resistant mice were observed (ns, TMCT). Our results demonstrate that obese mice had significantly higher body weights than overweight animals after 8 weeks on this moderately high fat diet (** *p* < 0.01, TMCT), whereas the differences of body weight between lean *vs*. obesity resistant mice were never statistically significant (ns, TMCT). 

Tumor size: Single tumors developed in every mice injected with tumor cells, and could be palpated a week after tumor cell injections. The association between mouse body weight and tumor progression was examined in tumor-bearing mice not subjected to any treatment (Figure 9D). Our results demonstrate that tumors from obese (OB), overweight (OW), obesity-resistant and lean mice are highly significantly different (** *p* < 0.01, two-way ANOVA). The largest tumors were seen in the obese mice followed by > overweight> obesity-resistant > lean (Figure 9D). Our data also demonstrate that obese mice at 4-weeks post tumor implantation have significantly larger tumors than lean mice (* *p* < 0.05, TMCT). At the same time point, however, tumors from overweight mice were not significantly different in size from tumors from obese mice (ns, overweight *vs.* obese tumor volume, TMCT). Tumors from obesity-resistant and lean mice were the smallest and were not significantly different in size (obesity-resistant *vs*. lean, ns, TMCT). 

To assess the *in vivo* role of leptin in tumor progression and macrophage recruitment to the mammary tumor microenvironment in DIO mice fed 33%HFD, we analyzed whether specifically blocking leptin signaling in mice with different body weights had any effect in tumor progression. To this aim, we treated lean, obesity-resistant, overweight and obese mice with one injection per week of the active pegylated leptin receptor antagonist peptide (PEG-LPrA2) or with vehicle (control untreated) during 4 weeks, and assessed tumor volume at euthanasia. Our results revealed that only in overweight (Figure 9E) and obese mice (Figure 9F) was treatment with the leptin antagonist peptide associated with a tendency for a reduction in tumor volume (ns Student’s* t*-test). We completed the *in vivo* studies with an analysis of serum levels of glucose, insulin, leptin and CCL2 in treated and untreated tumor bearers with different body weights, to investigate whether body weight and inhibition of leptin signaling might modulate the systemic levels of these important metabolic parameters. Using two-way ANOVA to analyze the results of leptin serum levels (Figure 9G), we revealed that treated *vs*. untreated groups were highly significantly different between themselves in their serum levels of leptin (*** *p* < 0.001). Two-way ANOVA also demonstrated that treated mice and non-treated mice with different body weights were also significantly different among themselves in their serum levels of leptin (*** *p* < 0.001). Multiple comparisons revealed that treatment with the leptin inhibitor significantly reduced the levels of serum leptin in obese mice (** *p* < 0.01, Sidak’s Multiple Comparison Test), and also that untreated obese mice have significantly higher levels of serum leptin compared with untreated obese-resistant mice (* *p* < 0.05, Tukey’s Multiple Comparison Test) (Figure 9G). Serum leptin levels could be mainly contributed by adipocytes from the tumor microenvironment and other adipose locations in obese mice containing larger fat masses. No significant differences were seen in the levels of glucose, insulin or CCL2 between these animals.

### 3.10. Evidences of Inflammation in the Mammary Tumor Microenvironment and Mammary Adipose Tissue of Obese Mice

**Figure 10:**
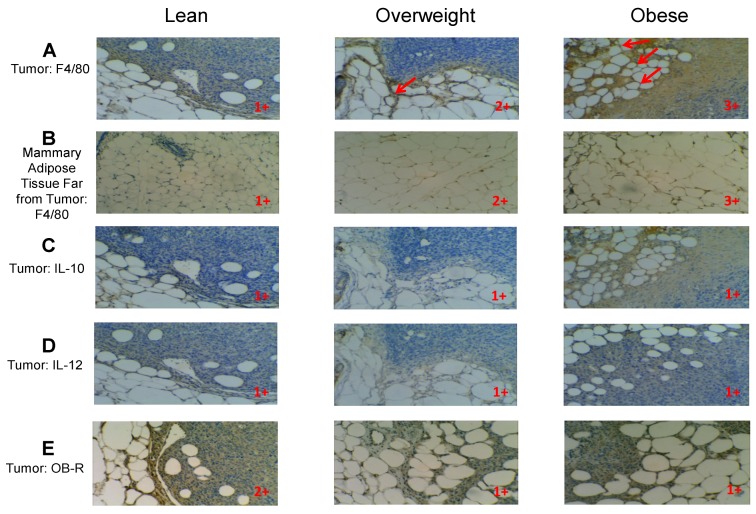
Mammary adipose tissue is inflammatory in obese mammary tumor-bearing females: comparison of mammary fat in the tumor microenvironment *vs*. mammary fat distant from the tumor. IHC was performed in sections of mammary tumors and in tumor-distal mammary adipose tissues of obese, overweight and lean C57 BL6 tumor bearer female mice. IHC- positive cells appear in brown (20× magnification in all the slides). (**A**) mammary tumor microenvironments of lean, overweight and obese mice showing F4/80^+^ macrophages and crown- like structures (CLS, depicted by red arrows). (**B**) Mammary adipose tissue biopsied distant from the tumor site showing F4/80^+^ macrophages in lean, overweight and obese tumor bearers. (**C**) IL-10^+^ cells in the tumor microenvironments of lean, overweight and obese tumor bearers. (**D**) IL-12+ cells in the tumor microenvironments of lean, overweight and obese tumor bearers. (**E**) Ob-R expression is reduced in the mammary tumor microenvironments of obese and overweight females. IHC was quantified by three independent observers and their average is shown in the figures: scores were 1+, 2+, 3+, where 1+ represents from 1–150 positive cells in the whole slide, 2+ represents 151–300 positive cells in the whole slide and 3+ represents more than 300 positive cells in the whole slide. Each experimental subgroup was comprised of ten (10) mice.

Visceral white adipose tissue in obese subjects is highly inflammatory, with hyperplastic and hypertrophic adipocytes, high numbers of M1 pro-inflammatory macrophages and crown like structures (CLS) where macrophages surround dying adipocytes [34]. We examined whether the mammary adipose tissue, an important component of the mammary tumor microenvironment, exhibits inflammatory features in obese mice. To assess inflammation, we determined adipocyte hypertrophy and examined the numbers of F4/80^+^ macrophages and the presence of CLS via IHC in the mammary tumor microenvironment of lean, overweight and obese mammary tumor-bearing female mice. We also studied mammary adipose tissue samples that were biopsied distant from the mammary tumor, to analyze whether this fat tissue is inflamed by itself in obesity. In addition, we analyzed the presence of M1 (IL-12^+^) and M2 (IL-10^+^) macrophages in the mammary tumor microenvironment. Finally, we studied whether body weight is associated with the expression of leptin receptor (Ob-R) in the mammary tumor microenvironment of mice with different weights. Our IHC results demonstrate that obese mice exhibit highest numbers of F4/80^+^ TAMs (and CLS) in the mammary tumor microenvironment, followed by overweight mice, as compared with lean mice, which exhibited the lowest numbers of F4/80^+^ macrophages and CLS (Figure 10A). Importantly, our results revealed for the first time that mammary adipose tissue *located far from the tumor* shows higher numbers of macrophages/adipocyte hypertrophy in obese mice than in overweight mice, with the lean mice showing the lowest numbers of macrophages/adipocytes hypertrophy (Figure 10B). We also found reduced numbers of F4/80^+^ macrophages/CLS in the mammary adipose tissue far from the tumor compared to the macrophages present in the tumor microenvironment within each weight category, strongly suggesting that tumor cell proximity increases F4/80^+^ macrophages and CLS in adipose tissue (Figure 10A,B). Figure 10C,D shows no differences in IL-10 or IL-12 expression in the mammary tumor microenvironments of mice with different body weights. Importantly, Figure 10E demonstrates that leptin-receptor expression decreases in the tumor microenvironment of overweight and obese mice, thus reproducing our *in vitro* data in macrophages exposed to leptin. In summary, our IHC results confirm our hypothesis that bAT in obese mammary tumor-bearing female mice (both within and distal to the tumor microenvironment) contains higher numbers of macrophages/CLS and hypertrophic adipocytes than bAT from lean tumor-bearing mice, indicating that the bAT is inflamed in mammary cancer in obesity. Also, our data suggest that the contact with tumor cells increases the amount of macrophages/CLS and adipocyte hypertrophy in the mammary fat. 

## 4. Discussion

White adipose tissue occurs basically in two different anatomical locations, visceral and subcutaneous, which differ in their capacities to become inflamed in obesity. Normally, visceral adipose tissue—but not subcutaneous—shows signs of chronic inflammation in obese individuals, characterized by elevated numbers of M1 macrophages, CLS and enlarged adipocytes [35]. The normal adult female breast is comprised largely of adipocytes and epithelial cells [36]. Interestingly bAT is considered subcutaneous fat due to its anatomical location in the breast. However, given the significant amount of adipose tissue that proportionally exists in the breast and its potential crosstalk with other cells of the parenchyma, it will be important to elucidate whether intermediate inflammatory changes do occur in the bAT of obese females, and particularly, of obese females with breast cancer. This obesity-induced bAT inflammation may have important implications in defining the tumor- promoting role of locally generated breast inflammation in breast cancer development. 

Until very recently, breast cancer was considered among the cancers not associated with chronic inflammation. Although probably still true in lean females who eat a healthy non inflammatory diet, this is not valid for obese females. In obese females, it is known that visceral obesity triggers systemic inflammation, enhancing cancer risk in general, including increasing risk of breast cancer and poor tumor prognosis. However, we reason that obesity may also locally impact bAT, generating local inflammation within the breast. Compared to other subcutaneous adipose tissues as the one underneath the skin, bAT encompasses a larger volume. This may allow adipocytes to expand their cellular size and their numbers, becoming hypertrophic and hyperplastic. The existence of different cell types within the breast cancer microenvironment—adipocytes included—facilitates crosstalk between them. We have shown that this interaction is capable of recruiting inflammatory cells and producing pro-inflammatory, tumor-promoting molecules in the DIO mouse. 

Macrophages, central cells in the inflammatory response, are the most frequent immune cell type in tumor microenvironments. TAMs assist tumor progression via different mechanisms and are considered a sign of poor tumor prognosis. These cells contribute to tumor initiation while they are pro-inflammatory and pro-mutagenic in tissues (M1) via their expression of ROI/RNI. They also participate in tumor progression when they acquire immunosuppressive and tissue repairing properties (M2), contributing to ECM remodeling, angiogenesis, invasion and metastasis. On the other hand, resident macrophages in lean adipose tissues are immunosuppressive M2, whereas they are pro-inflammatory M1 when they colonize obese AT, providing its inflammatory signature. 

We hypothesize that bAT from overweight/obese subjects is inflamed and promotes breast cancer development. This is due in large extent to the recruitment of macrophages to the bAT and tumor. For our studies, we used the E0771 mouse mammary tumor model, syngeneic to C57BL6 mice, which is the inbred mouse strain that is most sensitive to diet-induced obesity (DIO). In addition, E0771 is the only mammary tumor cell line syngeneic to C57BL6 mice; thus, the model used is the indicated one for our studies in obesity and mammary cancer in the mouse, given that genetic models of obesity in mice, such as the ob/ob or db/db mice, deficient in leptin or its receptor respectively, are resistant to mammary cancer [37]. To address our hypothesis we first carried out a series of *in vitro* studies focused on three main cell types of the mammary tumor microenvironment: adipocytes, mammary tumor cells and macrophages, and we studied the impact of their mutual interactions on macrophage recruitment and on tumor progression using *in vitro* co-cultures, and then conducted further studies in *in vivo* settings. 

In the *in vitro* studies, we centered our attention on three factors that could be produced in a paracrine fashion by these cells or their interactions, exerting monocyte chemotaxis by themselves or their combinations, and resulting in macrophage recruitment to the mammary tumor microenvironment. These paracrine factors are the adipokine leptin, the saturated fatty acid lauric acid and the chemokine CCL2. Adipocytes from obese fat tissues are known to produce high amounts of leptin and undergo lipolysis, releasing saturated fatty acids. Obese adipocytes express several pro-inflammatory chemokines, such as CCL2 (MCP-1). Mammary adipocytes from obese females may express all these molecules locally in the mammary gland, some of which could also be expressed by tumor cells. Co-culturing murine and human breast tumor cells with mature adipocytes, Dirat *et al.* [38] have shown that invasive cancer cells impact surrounding adipocytes, modifying their phenotype and biological features to become cancer-associated adipocytes, which alter the cancer cell’s characteristics leading to a more aggressive behavior. Their results strongly support the concept that adipocytes in the breast participate in breast cancer progression in a process orchestrated by tumor cells which may be amplified in obese women.

Leptin—the central regulator of satiety in the body—has been characterized as a pro-inflammatory, pro-angiogenic and proliferation-inducing adipokine essential to breast cancer [39]. The role of leptin signaling in enhancing the tumorigenic potential of tumor cells is well known [40], however whether leptin’s signaling in macrophages induces their recruitment to the breast tumor microenvironment or whether it activates in other ways macrophage’s pro-tumor functions, has not been thoroughly examined. Lauric acid is a saturated fatty acid released by adipocytes, especially upon obesity-induced lipolysis, and a ligand of TLR4, highly expressed by macrophages; the possible macrophage chemotactic capacity of this fatty acid had not been analyzed either. Chemokine CCL2 (MCP-1), one of the main chemo attractants for tissue monocytes/macrophages, might synergize with the other paracrine factors, further increasing macrophage recruitment to the tumor. Chronic inflammation is a potent inducer of many cancers, and expression of cytokines/chemokines within tumors has been correlated with poor prognosis [41]. Production of pro-inflammatory adipokines, cytokines, chemokines and saturated fatty acids by the different cell types within the breast tumor microenvironment could be exacerbated in obesity, resulting in an inflammatory situation in the breast of obese females. 

Our work demonstrates that adipocytes isolated *ex vivo* from visceral fat of obese female mice undergo lipolysis and release fatty acids. We observed that in the crosstalk of adipocytes with mammary tumor cells and macrophages—a setting that mimics the mammary/breast tumor microenvironment—lipolysis may be decreased, favoring inflammation. The decrease in FFA observed in the supernatants of the cell co-cultures may be explained mainly in two different ways: (1) an actual downregulation of lipolysis within adipocytes, which may result in increased accumulation of triglycerides and therefore in larger, hypertrophic adipocytes, characteristic of inflamed adipose tissue seen in the visceral fat of obese subjects; or (2) an increase in the uptake of FFA produced by adipocytes through lipolysis by tumor cells or macrophages. Either possibility, or the occurrence of both, is consistent with the reduced amount of FFA detected. Indeed, decreased lipolysis results in increased accumulation of triglycerides, and therefore in larger, hypertrophic adipocytes, characteristic of the inflamed obese adipose tissues of obese subjects. Alternatively, tumor cells and/or macrophages may increase uptake of fatty acids, as alternative sources of energy. We showed that *ex vivo* isolated adipocytes from obese adipose tissue are the major source of leptin production in our tumor model: we demonstrated that E0771 mammary tumor cells, in contrast to the majority of mammary/breast tumor cell lines, do not express leptin. Our results also demonstrate that co-culture with the tumor cells and/or macrophages decreases leptin production by adipocytes, suggesting that in the obese mammary tumor microenvironment of our E0771 DIO tumor model, leptin amounts may be lowered. Our results suggest that leptin is a marker of obesity rather than of adipocyte differentiation, since obese *ex vivo* isolated adipocytes but not differentiated 3T3-L1 adipocytes were the main producers of leptin in the system. Further studies with *ex vivo* adipocytes isolated from visceral adipose tissue of lean and obese mice will enable us to substantiate a broader generalization of this conclusion.

With respect to CCL2, our results demonstrate that the crosstalk between adipocytes from obese mice, macrophages and tumor cells—which is the setting that closely resembles the breast tumor microenvironment of obese females—is also the experimental condition that resulted in the highest amount of CCL2 detected among the cell co-cultures. Interestingly, as opposed to other tumor models where adipocytes are the main producers of CCL2 [42], in our E0771 tumor model, even adipocytes from obese adipose tissues produced negligible amounts of CCL2, and it is the E0771 mammary tumor cell line that is the main producer of this chemokine. We thus confirmed the expression of these different paracrine factors in the E0771 tumor model in conditions of obesity, and concluded that the setting that mimics the mammary tumor microenvironment (co-culture of the three cell types) is the one in general that favors the production of the highest concentrations of these paracrine molecules.

Recruitment of macrophages to the tumor microenvironment is a critical step in breast tumor inflammation. Analysis of monocyte chemotaxis by our three paracrine factors revealed that CCL2 is the major chemotactic factor, with lauric acid lacking such activity. With leptin, we detected a mild and non significant chemotaxis on THP1 monocytes at the lowest concentration used (3 ng/mL). Our results are in agreement with others who have reported that leptin is a chemoattractant at concentrations as low as 1 pg/mL with maximal effects at 1 ng/mL (which is why we did not see a significant chemotactic effect), and that when concentrations of 10–100 ng/mL are reached, leptin chemotaxis declined [43]. Also in agreement with Gruen *et al.* we found no evidence of additive chemotactic effects with the combination of leptin and CCL2 [43]. The crucial role of CCL2 in macrophage recruitment to the fat tissue in postmenopause has been reported in ovarectomized mice, where subcutaneous fat depots demonstrated elevated macrophage recruitment and increased expression of CCL2 compared to control mice, suggesting CCL2 may enhance inflammation in postmenopausal women [44]. CCL2 expression in tumors is correlated with higher histological grade breast cancer and is a significant indicator of early relapse [45]. Elevated CCL2 is also associated with TAMs infiltration in tumors [46]. In agreement with our results, it has been shown that obesity promotes breast cancer by CCL2-mediated macrophage recruitment [42]. We conclude that CCL2 is a major molecular player in our system. In terms of chemotaxis exerted by the cell supernatants, our results compellingly demonstrate that it is the co-culture of the three cell lines, resembling conditions in the breast tumor microenvironment, the setting that induces the highest monocyte recruitment. 

In the *in vitro* experiments which recreated the tumor microenvironment we used specific inhibitors for these paracrine factors, to reverse the specific functions identified: a novel antagonistic peptide—PEG-LPrA2—as a leptin signaling inhibitor which significantly delayed onset and decreased progression of mammary cancer in mice [29], or specific inhibitors for CCL2 and TLR4. Our experiments with the mixture of those specific inhibitors of the paracrine factors suggested that additional unidentified paracrine factors could be promoting monocyte chemotaxis to the obese mammary tumor microenvironment. To identify some of these new factors we used Luminex analysis. Our Luminex data revealed that adipocytes from obese adipose tissue, macrophages and mammary tumor cells, by themselves or interacting altogether, express numerous cytokines, chemokines and growth factors with known chemotactic, pro-inflammatory, pro-angiogenic and tumor-promoting activities, all of which may contribute to a mammary tumor microenvironment reflecting those different properties. Importantly, prevailing among all the different molecules that were identified in the co-culture of the three cell types was CCL2, followed by IP-10 (CXCL10) and IL-6, confirming the central role of these molecules in this tumor model. Cytokines secreted by the adipocytes and the tumor-associated immune cells and vasculature may also promote epithelial-to-mesenchymal transition (EMT). Because EMT plays a critical role in metastasis, obese adipocytes in the breast tissue can play a critical role in tumor progression which may explain why their presence is likely an underlying mediator of poor outcome in obese breast cancer patients [3,36].

To further investigate other plausible roles of leptin on macrophages, we examined whether acting in the tumor microenvironment or systemically, this adipokine might modulate macrophages M1/M2 activation. Activation with LPS (a TLR4 ligand and a prototypical activator of macrophages) was included to mimic the TLR4 activation that occurs in the tumor microenvironment through the action of endogenous ligands that are generated in tumors, such as heat shock proteins (HSP), high mobility group box 1 (HMGB1) and proteoglycans (Versican, Heparin sulfate, Hyaluronic Acid fragments). In contrast to what has been reported in the literature, where leptin is described as a pro-inflammatory adipokine, our results in peritoneal elicited macrophages clearly revealed that leptin decreases the constitutive expression of pro-inflammatory chemokine MIP-2 (CXCL2) and in a dose-response fashion downregulates the LPS or LPS+IFNγ-induced expression of the critical pro-inflammatory cytokines IL-12 and TNFα, as well as of the pro-inflammatory free radical nitric oxide (NO). In contrast, other investigators have found that leptin indirectly increases production of TNFα and other cytokines in monocytes [47]. Importantly, leptin induces the expression of inflammatory cytokines like IL-1 in tumor cells and non-tumor cells of the tumor stroma [29]; however we were not able to demonstrate IL-1 upregulation by leptin in macrophages, although IL-1 is a major inducer of IL-6 in a variety of cells. Interestingly, our data also demonstrate that leptin upregulates expression of the pro-inflammatory and tumor-promoting cytokine IL-6 upon LPS stimulation of these macrophages, although in similar conditions it does not modify expression of the main anti-inflammatory molecule IL-10. Surprisingly, IL-6 upregulation has been reported in macrophages from leptin-deficient ob/ob obese mice [48]. Together with CCL2 chemokine, IL-6 is the pro-inflammatory and tumor-promoting cytokine that we have seen consistently upregulated in our tumor model by the different cell types examined or their crosstalk. The pleiotropic cytokine IL-6 is known to play central roles in various types of cancers as a tumor growth factor, and is one of the main factors also responsible for cancer-induced cachexia. In breast cancer, it has recently been shown that IL-6 contributes to tumor progression [49,50].

Our work proves that leptin controls macrophage differentiation, as it downregulates the percentage of macrophages that express myeloid markers F4/80 and CD11b. We also reveal that leptin impairs protein expression of the central pro-inflammatory and tumor-promoting transcription factor NFκB in macrophages, yet strongly upregulates the expression of STAT3 and its active form (pSTAT3); both transcription factors operate in important tumor-promoting signaling pathways in macrophages and tumor cells. We showed that leptin does not regulate expression of pro-inflammatory STAT-1/pSTAT-1 yet increases IRAK-1 expression in macrophages, in agreement with others who have also reported leptin-induced increased expression of IRAK-1 in peritoneal macrophages [51]. In contrast, others have reported increased NFκB signaling in the stromal vascular fraction (SVF) of the mammary gland of high fat diet-induced obese mice, a fraction that these authors use as representative of the macrophages in bAT [22]. The SVF is actually comprised not only of macrophages but includes all the nonadipocyte cells in the adipose tissue, among them T and B cells, which also secrete cytokines, so this fraction may not identify exclusively macrophages. Leptin is reported to be anti-apoptotic in tumor cells [52], consistent with an upregulation of NFκB and STAT3 anti-apoptotic transcription factors. However, our results in leptin-treated macrophages reveal the opposite for NFκB although confirm the upregulation of pSTAT3 observed by these authors in tumor cells exposed to leptin. We have previously reported a similar contrasting effect between macrophages from tumor hosts and tumor cells [20] showing opposite expression patterns, e.g., decreased NFκB expression in TAMs and peritoneal macrophages as opposed to the constitutive NFκB upregulation that has been consistently reported in tumor cells [53]. Importantly, we provide the first report of a Notch protein modification induced by leptin in macrophages. Using the same E0771 mammary tumor cells and several human breast cancer cell lines we recently reported that when exposed to leptin, breast cancer cells express higher protein levels of Notch 3 [54], as we also found in the present study in macrophages exposed to low leptin concentrations. Interestingly, our results suggest that macrophages are more responsive than E0771 cells to the action of leptin on Notch 3 expression at lower levels of leptin, since 0.62 nM (10 ng/mL) leptin already upregulate Notch 3 in macrophages whereas a concentration of 6.2 nM (100 ng/mL), 10-fold higher, is required in E0771 cells to see a consistent increase in Notch 3 expression in these tumor cells [54]. Alterations of the Notch signaling pathway can lead to a variety of disorders, including cancer. We have reported the existence of a crosstalk between oncogenic signaling pathways in breast tumor cells in humans and mice triggered by leptin, IL-1 and Notch (NILCO) critical to leptin-induced breast cancer promotion [31].

We examined whether the presence of leptin might modulate the expression of its own receptor in macrophages. Macrophages, as other cells susceptible to leptin’s action, express two main forms of the leptin receptor, *i.e.*, the long, full-length form (Ob-Rb) and the short form (Ob-Ra), both involved in leptin signaling [55]. We report for the first time that leptin profoundly downregulates in a clear dose-response fashion both forms of its own receptor in peritoneal macrophages as well as in TAMs. As we previously discussed, leptin’s concentration in the *in vitro*-simulated murine obese mammary tumor microenvironment is decreased after adipocytes crosstalk with E0771 tumor cells (which do not produce leptin themselves) and with macrophages. Thus, leptin’s actual concentrations in the tumor microenvironment of the E0771 obese mammary tumor model may not be as high as the ones which experimentally downregulated its receptor in macrophages. In any case, it is interesting to note that the effects that we have described are induced by leptin in macrophages occur despite the downregulation of its receptor after exposure to leptin. Importantly, the chemotactic activity of leptin in THP1 monoctytic cells requires the expression of the long and the short forms of the leptin receptor [43,56], so this leptin receptor downregulation is not seen in monocytes. The protein downregulation of leptin receptor that we report in both types of macrophages was observed after 20 h stimulation with leptin. Signaling events occur in minutes, after which the receptor may be downregulated by mechanisms that remain to be elucidated and that are beyond the aims of this study. 

Our animal experiments with DIO and lean C57BL6 mice bearing E0771 mammary tumors were specifically designed to examine *in vivo* the role of leptin in TAMs recruitment to the mammary tumors and in tumor progression, as well as to assess the association of body weight with bAT inflammation in mice fed a HFD that results in obese and overweight mice. Mice were fed with a 33% Kcal/fat HFD. This diet was selected because it reproduces the current human Western diet. Mice fed this diet, as with humans, have a varied response in that some become obese, others overweight, and others that remain resistant to obesity. We also had animals fed a 10%Kcal/fat which remained as lean controls. Our results confirm that obesity significantly contributes to tumor size, with the obese mice bearing the largest tumors and the lean animals bearing the smaller ones. Interestingly, tumor sizes were comparable between overweight and obese mice, suggesting that being overweight already involves a level of tumor aggressiveness and progression similar to the one observed in obese mice. Using a different HFD (60%Kcal/fat) in the same E0771 tumor model we recently reported that obesity tends to positively increase the detection rate of mammary cancer in DIO mice [54]. Injecting our mice with a designated treatment scheme (a single dose of 50 µL/100 µM i.v. in tail vein per week) of the same leptin receptor antagonist peptide (PEG-LPrA2) that was used in our *in vitro* chemotaxis experiments at other appropriate concentrations, our *in vivo* results demonstrate a non significant tendency of this peptide to reduce tumor sizes in obese and overweight mice. A significant increase in serum leptin levels was observed in obese but not in overweight mice when compared with obese-resistant mice. Interestingly, treatment with the leptin inhibitor significantly reduced those elevated serum leptin levels in obese mice only. Corroborating our *in vitro* experiments mimicking the E0771 tumor microenvironment of obese mice, where we demonstrated that leptin is not produced by the tumor cells or macrophages but by obese adipocytes, overall our *in vivo* results indicate that in this DIO tumor model, when mice are fed 33% HFD, leptin inhibition at the doses used was not enough to significantly reduce tumor growth, particularly when the tumor-promoting factors CCL2 and IL-6 are present. Interestingly, recent *in vivo* studies from our group using the same mammary tumor model but a different HFD (60% instead of 33%, as we used here), and a more aggressive treatment scheme with the peptide inhibitor (two injections per week instead of one), showed significant results in tumor regression, suggesting a more relevant role for leptin in the same tumor model in mice fed a different diet and treated more aggressively with the leptin inhibitor [54]. Taken together, these results illustrate how a different diet can regulate the biological characteristics of a tumor, making our conclusions in the present paper relevant to the diet and experimental conditions we used here. 

We capitalized on the opportunity offered by the 33% HFD which generates obese and overweight mice to examine the association between body weight and bAT inflammation. We analyzed bAT within the mammary tumor microenvironment as well as in areas distal from the tumor in the mammary gland, to investigate whether body weight is related to bAT inflammation in the tumor microenvironment and also distant from it. Our results compellingly demonstrate that obese mice have higher numbers of macrophages and CLS than overweight mice in their bAT, and that lean animals have the least, strongly indicating that body weight is associated with bAT inflammation in the tumor microenvironment. Cinti *et al.* [35] reported a high occurrence of “crown-like structures” (CLS) in obese white fat of humans and mice. These CLS are histological configurations where dying adipocytes are surrounded by macrophages; these structures are considered a hallmark of chronic inflammation. More recently Subbaramaiah *et al.* [22] have demonstrated an association between CLS with NFκB activation, increased levels of pro-inflammatory mediators (TNF-α, IL-1β, Cox-2) and elevated levels of aromatase expression/activity in the breast and visceral adipose tissues of obese mice and women. We additionally showed that bAT from obese mice is accompanied by high numbers of isolated macrophages and hypertrophic adipocytes, as additional parameters of adipose tissue inflammation that should be considered. Importantly, we also report here that inflammation in the bAT distal from the tumor microenvironment is also associated with body weight. Our results reveal that bAT located far from the tumor contains higher numbers of macrophages/CLS and adipocyte hypertrophy in obese mice than in overweight mice, with the lean mice showing the lowest numbers of macrophages/adipocytes hypertrophy, an *in vivo* observation that we report here for the first time. Interestingly, we verified that within each weight group there is higher inflammation in the bAT from the tumor microenvironment than in the tumor-distal bAT, strongly indicating that the contact with tumor cells increases bAT inflammation. We could not see any differences in the expression of IL-12 (M1) *vs*. IL-10 (M2) macrophages in the mammary tumor microenvironments of obese, overweight or lean mice, suggesting that obesity is not particularly related to M1 or M2 macrophages in the mammary tumor microenvironment. This result was anticipated, since although obese visceral adipose tissues are colonized mainly by M1 macrophages, TAMs in tumor microenvironments have been reported to be principally M2 or mixtures of M1 and M2 [19,20]. Thus, bAT within a tumor microenvironment from an obese female should be expected to show mixtures of M1 and M2 macrophages. We were able to consistently confirm *in vivo* in the tumor microenvironment our *ex vivo* results where we found Ob-R downregulation in peritoneal macrophages and TAMs upon their pre-treatment with leptin. Our IHC results show that leptin-receptor expression decreases in the tumor microenvironment of overweight and obese mice. In summary, our work confirms our hypothesis that bAT within the tumor microenvironment of obese mammary tumor bearing females contains higher numbers of macrophages/CLS and hypertrophic adipocytes than bAT from lean tumor-bearing mice, indicating that the bAT is more inflamed in breast cancer in obesity. Also, our data demonstrate that the bAT by itself (distant from the tumor microenvironment) is more inflamed in obese than in lean mice, strongly suggesting that the contact with tumor cells increases the amount of macrophages/CLS in the breast fat. 

## 5. Concluding Remarks and Future Directions

Using *in vitro* and *in vivo* settings with a syngeneic mouse mammary tumor model of DIO, we have attempted to mimic the situation in human breast cancer in the context of obesity. In the effort to extend our results to the human situation, it is important to consider that all the complex facets of human obesity and breast cancer are beyond this mock characterization, and that this is an oversimplified although important starting point to use as reference. Our work demonstrates that crosstalk of adipocytes from obese adipose tissue with mammary tumor cells and macrophages contributes to monocyte recruitment and to the expression of diverse molecules that are chemotactic to macrophages and to other inflammatory cells in the tumor microenvironment. In addition to confirming the presence of leptin, fatty acids and CCL2, we identified several other paracrine molecules produced by the crosstalk among the three cell types that also exhibit chemotactic, pro-angiogenic, pro-inflammatory, and tumor-promoting functions. We show that CCL2 is the main molecule produced in this DIO tumor model with chemotactic and tumor-promoting activities, followed by the chemokine IP-10 (CXCL10) and by the pro-tumor pro-inflammatory cytokine IL-6. Centering our interest in the role of leptin in this E0771 mammary *in vitro/in vivo* tumor model, we found that, contrary to most breast/mammary tumor cell lines, E0771 cells do not express leptin. We could not demonstrate strong monocytic chemotactic properties for leptin either, although its interaction with CCL2 and lauric acid resulted in enhanced chemotactic properties for monocytes. We provide for the first time evidence of the pleiotropic effects of leptin on macrophages: leptin regulates macrophage M1/M2 activation, given it downregulates pro-inflammatory molecules IL-12, nitric oxide, TNFα and MIP-2 (CXCL2). Leptin however upregulates pro-inflammatory and pro-tumor IL-6 yet does not alter anti-inflammatory IL-10 expression. We confirm it modulates differentiation and expression of important tumor-promoting signaling pathways in macrophages, such as NFκB, STAT3, Notch 3 and IRAK1. Our study with diet-induced obese (DIO) mice using the E0771 mammary tumor model confirms that obese female mice have larger mammary tumors than lean mice, and that their mammary tumor microenvironments contain more TAMs, CLS and hypertrophic adipocytes. Furthermore, we demonstrate that the mammary fat close to the tumor contains more macrophages and CLS than the one far from the tumor, but that this mammary fat distal from the tumor is inflamed in obesity as well, yet to a lesser degree that when it is near to the tumor. Treatment with the PEG-LPrA2 leptin antagonist was associated with a non significant tendency to reduce tumor progression in obese and overweight mice but not in lean ones. Obese mice express higher serum levels of leptin, and inhibition of leptin signaling significantly reduced these high serum leptin levels in obese mice. At this same dose, no association between treatment with the leptin antagonist peptide PEG-LPrA2 and TAM or CLS in tumor or fat was observed. Importantly, we found that peritoneal macrophages and TAMs downregulate the expression of the long and short forms of leptin receptor. 

Based on our results we suggest that in this E0771 mammary tumor model in DIO mice fed a 33% HFD that resembles human Western diet, CCL2 plays a more critical role than leptin in macrophage recruitment to the tumor microenvironment and in tumor progression, although leptin regulates many important macrophage functions. Therefore, we plan to investigate whether administering a mixture of leptin inhibitor together with a CCL2 inhibitor to tumor bearers will result in a better approach to reduce tumor progression and adipose tissue inflammation in this tumor model with DIO mice. Inhibition of inflammation can limit macrophage infiltration during tumor development. Given that tumorigenesis is almost entirely prevented in mice following macrophage ablation, the use of molecules that specifically target and eliminate macrophages, such as Clodrolip [57], could have substantial therapeutic benefits in breast cancer treatment, especially if targeted to the tumor microenvironment. This approach could be combined with treatments with CCL2 blocking antibody and the anti-leptin signaling peptide in obese tumor-bearing mice, aiming to inhibit macrophage recruitment/activity in the mammary tumor microenvironment. Obesity is one of the main preventable causes of cancer. Weight control is the primary prevention strategy for breast cancer, given the major role of obesity in modulating systemic and breast adipose tissue inflammation. It is possible that agents that suppress the obesity-inflammation relationship, such as those that modulate macrophages, will have a role in inhibiting breast tumor progression.

## Supplementary Files

Supplementary File 1
