# Supplementary material for: Paracrine Interactions between Adipocytes and Tumor Cells Recruit and Modify Macrophages to the Mammary Tumor Microenvironment: The Role of Obesity and Inflammation in Breast Adipose Tissue
Source: Cancers (Basel). 2015 Jan 15;7(1):143–78. doi: 10.3390/cancers7010143 (PMC4381255; doi:10.3390/cancers7010143)
Supplement: Supplementary File 1 [file cancers-07-00143-s001.pdf]

Paracrine Interactions between Adipocytes and Tumor Cells Recruit and Modify Macrophages to the Mammary Tumor Microenvironment: the Role of Obesity and Inflammation in Breast Adipose Tissue

Supplementary Table of Figure 1. Statistical analysis of Free Fatty Acid (FFA) production by adipocytes, macrophages and mammary tumor cells.

One way ANOVA: * $p < 0.05$

Tukey's multiple comparisons.

ns $p > 0.05$ * $p < 0.05$ ** $p < 0.01$ *** $p < 0.001$ **** $p < 0.0001$

**Comparison**	**Significance**
3T3-L1 *vs*. 3T3-L1/N-PEMs	ns
3T3-L1 *vs*. 3T3-L1/E0771	ns
3T3-L1 *vs*. 3T3-L1/N-PEMs/E0771	*
3T3-L1/N-PEMs *vs*. 3T3-L1/E0771	ns
3T3-L1/N-PEMs *vs*. 3T3-L1/N-PEMs/E0771	ns
3T3-L1/E0771 *vs*. 3T3-L1/N-PEMs/E0771	ns

Supplementary Tables of Figure 2. Statistical analysis of leptin production by adipocytes, macrophages and mammary tumor cells.

<u>A</u>

One way ANOVA: *** $P < 0.001$

Tukey's multiple comparisons.

ns $p > 0.05$ * $p < 0.05$ ** $p < 0.01$ *** $p < 0.001$ **** $p < 0.0001$

**Comparison**	**Significance**
3T3 *vs*. 3T3/N-PEM	**
3T3 *vs*. 3T3/E0771	ns
3T3 *vs*. 3T3/N-PEM/E0771	*
3T3/N-PEM *vs*. 3T3/E0771	**
3T3/N-PEM *vs*. 3T3/N-PEM/E0771	***
3T3/E0771 *vs*. 3T3/N-PEM/E0771	**

<u>B</u>

One way ANOVA: **** $p < 0.0001$

Tukey's multiple comparisons.

ns $p > 0.05$ *$p < 0.05$ **$p < 0.01$ ***$p < 0.001$ ****$p < 0.0001$

**Comparison**	**Significance**
Adipocytes (*ex vivo*) *vs*. Adipocytes/N-PEM	**
Adipocytes (*ex vivo*) *vs*. Adipocytes/E0771	****
Adipocytes (*ex vivo*) *vs*. Adipocytes/N-PEM/E0771	**
Adipocytes/N-PEM *vs*. Adipocytes/E0771	****
Adipocytes/N-PEM *vs*. Adipocytes/N-PEM/E0771	ns
Adipocytes/E0771 *vs*. Adipocytes/N-PEM/E0771	****

Supplementary Tables of Figure 3. Statistical analysis of CCL2 production by adipocytes, macrophages and mammary tumor cells.

A

One way ANOVA: **** $p < 0.0001$

Tukey's multiple comparisons.

ns $p > 0.05$ * $p < 0.05$ ** $p < 0.01$ *** $p < 0.001$ **** $p < 0.0001$

**Comparison**	**Significance**
3T3 *vs*. 3T3/N-PEM	ns
3T3 *vs*. 3T3/E0771	****
3T3 *vs*. 3T3/N-PEM/E0771	****
3T3 *vs*. E0771	****
3T3/N-PEM *vs*. 3T3/E0771	****
3T3/N-PEM *vs*. 3T3/N-PEM/E0771	****
3T3/N-PEM *vs*. E0771	****
3T3/E0771 *vs*. 3T3/N-PEM/E0771	ns
3T3/E0771 *vs*. E0771	ns
3T3/N-PEM/E0771 *vs*. E0771	ns

B

One way ANOVA: **** $p < 0.0001$

Tukey's multiple comparisons.

ns $p > 0.05$ * $p < 0.05$ ** $p < 0.01$ *** $p < 0.001$ **** $p < 0.0001$

**Comparison**	**Significance**
Adipocytes (*ex vivo*) *vs*. Adipocytes/N-PEM	ns
Adipocytes (*ex vivo*) *vs*. Adipocytes/E0771	***
Adipocytes (*ex vivo*) *vs*. Adipoc/N-PEM/E0771	****
Adipocytes (*ex vivo*) *vs*. E0771	***
Adipocytes/N-PEM *vs*. Adipocytes/E0771	**

Adipocytes/N-PEM *vs*. Adipoc/N-PEM/E0771	****
Adipocytes/N-PEM *vs*. E0771	***
Adipocytes/E0771 *vs*. Adipoc/N-PEM/E0771	**
Adipocytes/E0771 *vs*. E0771	ns
Adipoc/N-PEM/E0771 *vs*. E0771	*

Supplementary Tables of Figure 4. Statistical analysis of migration experiments.

A1

One way ANOVA: *** $p < 0.001$

Tukey's multiple comparisons.

ns $p > 0.05$ * $p < 0.05$ ** $p < 0.01$ *** $p < 0.001$ **** $p < 0.0001$

**Comparison**	**Significance**
Medium *vs*. CCL2 (10 ng/mL)	*
Medium *vs*. CCL2 (10 ng/mL)/anti-CCL2	ns
Medium *vs*. CCL2 (50 ng/mL)	***
Medium *vs*. CCL2 (50 ng/mL)/anti-CCL2	*
CCL2 (10 ng/mL) *vs*. CCL2 (10 ng/mL)/anti-CCL2	ns
CCL2 (10 ng/mL) *vs*. CCL2 (50 ng/mL)	**
CCL2 (10 ng/mL) *vs*. CCL2 (50 ng/mL)/anti-CCL2	ns
CCL2 (10 ng/mL)/anti-CCL2 *vs*. CCL2 (50 ng/mL)	**
CCL2 (10 ng/mL)/anti-CCL2 *vs*. CCL2 (50 ng/mL)/anti-CCL2	ns
CCL2 (50 ng/mL) *vs*. CCL2 (50 ng/mL)/anti-CCL2	**

A2

One way ANOVA: *** $p < 0.001$

Tukey's multiple comparisons.

ns $p > 0.05$ * $p < 0.05$ ** $p < 0.01$ *** $p < 0.001$ **** $p < 0.0001$

**Comparison**	**Significance**
Medium *vs*. CCL2 (50 ng/mL)	***
Medium *vs*. Leptin (3 ng/mL)	ns
Medium *vs*. Leptin (100 ng/mL)	ns
Medium *vs*. Leptin (500 ng/mL)	ns
CCL2 (50 ng/mL) *vs*. Leptin (3 ng/mL)	**
CCL2 (50 ng/mL) *vs*. Leptin (100 ng/mL)	***
CCL2 (50 ng/mL) *vs*. Leptin (500 ng/mL)	***
Leptin (3 ng/mL) *vs*. Leptin (100 ng/mL)	ns
Leptin (3 ng/mL) *vs*. Leptin (500 ng/mL)	ns
Leptin (100 ng/mL) *vs*. Leptin (500 ng/mL)	ns

A3

One way ANOVA: **** $p < 0.0001$

Tukey's multiple comparisons.

ns $p > 0.05$ * $p < 0.05$ ** $p < 0.01$ *** $p < 0.001$ **** $p < 0.0001$

**Comparison**	**Significance**
Medium *vs*. CCL2 (50 ng/mL)	****
Medium *vs*. LA (2.5 mM)	ns
Medium *vs*. LA (5 mM)	ns
Medium *vs*. LA (10 mM)	ns
Medium *vs*. LA (100 mM)	ns
CCL2 (50 ng/mL) *vs*. LA (2.5 mM)	****
CCL2 (50 ng/mL) *vs*. LA (5 mM)	****
CCL2 (50 ng/mL) *vs*. LA (10 mM)	****
CCL2 (50 ng/mL) *vs*. LA (100 mM)	****
LA (2.5 mM) *vs*. LA (5 mM)	ns
LA (2.5 mM) *vs*. LA (10 mM)	ns
LA (2.5 mM) *vs*. LA (100 mM)	ns
LA (5 mM) *vs*. LA (10 mM)	ns
LA (5 mM) *vs*. LA (100 mM)	ns
LA (10 mM) *vs*. LA (100 mM)	ns

B

One way ANOVA: ** $p < 0.01$

Tukey's multiple comparisons.

ns $p > 0.05$ * $p < 0.05$ ** $p < 0.01$ *** $p < 0.001$ **** $p < 0.0001$

**Comparison**	**Significance**
Medium *vs*. CCL2	*
Medium *vs*. CCL2/Leptin	*
Medium *vs*. CCL2/Leptin/LA	*
Medium *vs*. Leptin/LA	ns
CCL2 *vs*. CCL2/Leptin	ns
CCL2 *vs*. CCL2/Leptin/LA	ns
CCL2 *vs*. Leptin/LA	*
CCL2/Leptin *vs*. CCL2/Leptin/LA	ns
CCL2/Leptin *vs*. Leptin/LA	*
CCL2/Leptin/LA *vs*. Leptin/LA	*

C1

One way ANOVA: **** $p < 0.0001$

Tukey's multiple comparisons.

ns $p > 0.05$ * $p < 0.05$ ** $p < 0.01$ *** $p < 0.001$ **** $p < 0.0001$

**Comparison**	**Significance**
Medium *vs.* CCL2 (50 ng/mL)	****
Medium *vs.* CCL2 (50 ng/mL)/anti-CCL2	*
Medium *vs.* 3T3-L1 Supernatant	****
Medium *vs.* Adipoc. (*ex vivo*) Supernat	****
Medium *vs.* N-PEM Supernat.	ns
Medium *vs.* E0771 Supernat.	****
Medium *vs.* E0771/anti-CCL2	****
CCL2 (50 ng/mL) *vs.* CCL2 (50 ng/mL)/anti-CCL2	****
CCL2 (50 ng/mL) *vs.* 3T3-L1 Supernatant	****
CCL2 (50 ng/mL) *vs.* Adipoc. (*ex vivo*) Supernat	*
CCL2 (50 ng/mL) *vs.* N-PEM Supernat.	****
CCL2 (50 ng/mL) *vs.* E0771 Supernat.	ns
CCL2 (50 ng/mL) *vs.* E0771/anti-CCL2	ns
CCL2 (50 ng/mL)/anti-CCL2 *vs.* 3T3-L1 Supernatant	***
CCL2 (50 ng/mL)/anti-CCL2 *vs.* Adipoc. (*ex vivo*) Supernat	****
CCL2 (50 ng/mL)/anti-CCL2 *vs.* N-PEM Supernat.	ns
CCL2 (50 ng/mL)/anti-CCL2 *vs.* E0771 Supernat.	****
CCL2 (50 ng/mL)/anti-CCL2 *vs.* E0771/anti-CCL2	****
3T3-L1 Supernatant *vs.* Adipoc. (*ex vivo*) Supernat	****
3T3-L1 Supernatant *vs.* N-PEM Supernat.	****
3T3-L1 Supernatant *vs.* E0771 Supernat.	****
3T3-L1 Supernatant *vs.* E0771/anti-CCL2	****
Adipoc. (*ex vivo*) Supernat *vs.* N-PEM Supernat.	****
Adipoc. (*ex vivo*) Supernat *vs.* E0771 Supernat.	ns
Adipoc. (*ex vivo*) Supernat *vs.* E0771/anti-CCL2	ns
N-PEM Supernat. *vs.* E0771 Supernat.	****
N-PEM Supernat. *vs.* E0771/anti-CCL2	****
E0771 Supernat. *vs.* E0771/anti-CCL2	ns

C2

One way ANOVA: **** $p < 0.0001$

Tukey's multiple comparisons.

ns $P > 0.05$ * $p < 0.05$ ** $p < 0.01$ *** $p < 0.001$ **** $p < 0.0001$

**Comparison**	**Significance**
Medium *vs.* CCL2 (50 ng/mL)	****
Medium *vs.* Adipoc./E0771	****
Medium *vs.* Adipoc./E0771/N-PEM	****
Medium *vs.* 3T3-L1/E0771/N-PEM	***
CCL2 (50 ng/mL) *vs.* Adipoc./E0771	ns
CCL2 (50 ng/mL) *vs.* Adipoc./E0771/N-PEM	ns
CCL2 (50 ng/mL) *vs.* 3T3-L1/E0771/N-PEM	ns
Adipoc./E0771 *vs.* Adipoc./E0771/N-PEM	ns
Adipoc./E0771 *vs.* 3T3-L1/E0771/N-PEM	*
Adipoc./E0771/N-PEM *vs.* 3T3-L1/E0771/N-PEM	ns

C3

One way ANOVA: **** $p < 0.0001$

Tukey's multiple comparisons.

ns $p > 0.05$ * $p < 0.05$ ** $p < 0.01$ *** $p < 0.001$ **** $p < 0.0001$

**Comparison**	**Significance**
Medium *vs.* CCL2 (50 ng/mL)	****
Medium *vs.* CCL2 (50 ng/mL)/anti-CCL2	*
Medium *vs.* Adipoc/E0771	****
Medium *vs.* Adipoc/E0771/anti-CCL2	****
Medium *vs.* Adipoc/E0771/inhib mix	****
Medium *vs.* Adipoc/E0771/N-PEM	****
Medium *vs.* Adipoc/E0771/N-PEM/anti-CCL2	****
Medium *vs.* Adipoc/E0771/ N-PEM/inhib mix	****
CCL2 (50 ng/mL) *vs.* CCL2 (50 ng/mL)/anti-CCL2	**
CCL2 (50 ng/mL) *vs.* Adipoc/E0771	ns
CCL2 (50 ng/mL) *vs.* Adipoc/E0771/anti-CCL2	ns
CCL2 (50 ng/mL) *vs.* Adipoc/E0771/inhib mix	ns
CCL2 (50 ng/mL) *vs.* Adipoc/E0771/N-PEM	ns
CCL2 (50 ng/mL) *vs.* Adipoc/E0771/N-PEM/anti-CCL2	ns
CCL2 (50 ng/mL) *vs.* Adipoc/E0771/ N-PEM/inhib mix	ns
CCL2 (50 ng/mL)/anti-CCL2 *vs.* Adipoc/E0771	***
CCL2 (50 ng/mL)/anti-CCL2 *vs.* Adipoc/E0771/anti-CCL2	**
CCL2 (50 ng/mL)/anti-CCL2 *vs.* Adipoc/E0771/inhib mix	**
CCL2 (50 ng/mL)/anti-CCL2 *vs.* Adipoc/E0771/N-PEM	***
CCL2 (50 ng/mL)/anti-CCL2 *vs.* Adipoc/E0771/ N-PEM/anti-CCL2	***
CCL2 (50 ng/mL)/anti-CCL2 *vs.* Adipoc/E0771/ N-PEM/inhib mix	**
Adipoc/E0771 *vs.* Adipoc/E0771/anti-CCL2	ns
Adipoc/E0771 *vs.* Adipoc/E0771/inhib mix	ns

Adipoc/E0771 *vs.* Adipoc/E0771/N-PEM	ns
Adipoc/E0771 *vs.* Adipoc/ E0771/N-PEM/anti-CCL2	ns
Adipoc/E0771 *vs.* Adipoc/E0771/ N-PEM/inhib mix	ns
Adipoc/E0771/anti-CCL2 *vs.* Adipoc/E0771/inhib mix	ns
Adipoc/E0771/anti-CCL2 *vs.* Adipoc/E0771/N-PEM	ns
Adipoc/E0771/anti-CCL2 *vs.* Adipoc/ E0771/ N-PEM/anti-CCL2	ns
Adipoc/E0771/anti-CCL2 *vs.* Adipoc/E0771/ N-PEM/inhib mix	ns
Adipoc/E0771/inhib mix *vs.* Adipoc/E0771/N-PEM	ns
Adipoc/E0771/inhib mix *vs.* Adipoc/ E0771/ N-PEM/anti-CCL2	ns
Adipoc/E0771/inhib mix *vs.* Adipoc/E0771/ N-PEM/inhib mix	ns
Adipoc/E0771/N-PEM *vs.* Adipoc/ E0771/ N-PEM/anti-CCL2	ns
Adipoc/E0771/N-PEM *vs.* Adipoc/E0771/ N-PEM/inhib mix	ns
Adipoc/ E0771/ N-PEM/anti-CCL2 *vs.* Adipoc/E0771/ N-PEM/inhib mix	ns

Supplementary Tables of Figure 6. Statistical analysis of leptin as a regulator of cytokine and chemokine expression in macrophages.

A: IL-12p70

One way ANOVA: **** $p < 0.0001$

Tukey's multiple comparisons.

ns $p > 0.05$ * $p < 0.05$ ** $p < 0.01$ *** $p < 0.001$ **** $p < 0.0001$

**Comparison**	**Significance**
Control *vs.* LPS	ns
Control *vs.* LPS+IFN	****
Control *vs.* Lep 10	ns
Control *vs.* LPS+Lep 10	ns
Control *vs.* LPS+IFN+Lep 10	**
Control *vs.* Lep 100	ns
Control *vs.* LPS+Lep 100	ns
Control *vs.* LPS+IFN+Lep 100	ns
LPS *vs.* LPS+IFN	****
LPS *vs.* Lep 10	ns
LPS *vs.* LPS+Lep 10	ns
LPS *vs.* LPS+IFN+Lep 10	**
LPS *vs.* Lep 100	ns
LPS *vs.* LPS+Lep 100	ns
LPS *vs.* LPS+IFN+Lep 100	ns
LPS+IFN *vs.* Lep 10	****
LPS+IFN *vs.* LPS+Lep 10	****
LPS+IFN *vs.* LPS+IFN+Lep 10	**
LPS+IFN *vs.* Lep 100	****
LPS+IFN *vs.* LPS+Lep 100	****

LPS+IFN *vs.* LPS+IFN+Lep 100	****
Lep 10 *vs.* LPS+Lep 10	ns
Lep 10 *vs.* LPS+IFN+Lep 10	**
Lep 10 *vs.* Lep 100	ns
Lep 10 *vs.* LPS+Lep 100	ns
Lep 10 *vs.* LPS+IFN+Lep 100	ns
LPS+Lep 10 *vs.* LPS+IFN+Lep 10	**
LPS+Lep 10 *vs.* Lep 100	ns
LPS+Lep 10 *vs.* LPS+Lep 100	ns
LPS+Lep 10 *vs.* LPS+IFN+Lep 100	ns
LPS+IFN+Lep 10 *vs.* Lep 100	**
LPS+IFN+Lep 10 *vs.* LPS+Lep 100	**
LPS+IFN+Lep 10 *vs.* LPS+IFN+Lep 100	*
Lep 100 *vs.* LPS+Lep 100	ns
Lep 100 *vs.* LPS+IFN+Lep 100	ns
LPS+Lep 100 *vs.* LPS+IFN+Lep 100	ns

A: IL-10

One way ANOVA: **** $p < 0.0001$

Tukey's multiple comparisons.

ns $p > 0.05$ * $p < 0.05$ ** $p < 0.01$ *** $p < 0.001$ **** $p < 0.0001$

**Comparison**	**Significance**
Control *vs.* LPS	****
Control *vs.* LPS+IFN	ns
Control *vs.* Lep 10	ns
Control *vs.* LPS+Lep 10	****
Control *vs.* LPS+IFN+Lep 10	ns
Control *vs.* Lep 100	ns
Control *vs.* LPS+Lep 100	****
Control *vs.* LPS+IFN+Lep 100	ns
LPS *vs.* LPS+IFN	****
LPS *vs.* Lep 10	****
LPS *vs.* LPS+Lep 10	ns
LPS *vs.* LPS+IFN+Lep 10	****
LPS *vs.* Lep 100	****
LPS *vs.* LPS+Lep 100	ns
LPS *vs.* LPS+IFN+Lep 100	****
LPS+IFN *vs.* Lep 10	ns
LPS+IFN *vs.* LPS+Lep 10	****
LPS+IFN *vs.* LPS+IFN+Lep 10	ns
LPS+IFN *vs.* Lep 100	ns
LPS+IFN *vs.* LPS+Lep 100	****

LPS+IFN *vs.* LPS+IFN+Lep 100	ns
Lep 10 *vs.* LPS+Lep 10	****
Lep 10 *vs.* LPS+IFN+Lep 10	ns
Lep 10 *vs.* Lep 100	ns
Lep 10 *vs.* LPS+Lep 100	****
Lep 10 *vs.* LPS+IFN+Lep 100	ns
LPS+Lep 10 *vs.* LPS+IFN+Lep 10	****
LPS+Lep 10 *vs.* Lep 100	****
LPS+Lep 10 *vs.* LPS+Lep 100	ns
LPS+Lep 10 *vs.* LPS+IFN+Lep 100	****
LPS+IFN+Lep 10 *vs.* Lep 100	ns
LPS+IFN+Lep 10 *vs.* LPS+Lep 100	****
LPS+IFN+Lep 10 *vs.* LPS+IFN+Lep 100	ns
Lep 100 *vs.* LPS+Lep 100	****
Lep 100 *vs.* LPS+IFN+Lep 100	ns
LPS+Lep 100 *vs.* LPS+IFN+Lep 100	****

<u>A: Nitric Oxide</u>

One way ANOVA: **** $p < 0.0001$

Tukey's multiple comparisons.

ns $p > 0.05$ * $p < 0.05$ ** $p < 0.01$ *** $p < 0.001$ **** $p < 0.0001$

**Comparison**	**Significance**
Control *vs.* LPS	ns
Control *vs.* LPS+IFN	****
Control *vs.* Lep 10	ns
Control *vs.* LPS+Lep 10	ns
Control *vs.* LPS+IFN+Lep 10	****
Control *vs.* Lep 100	ns
Control *vs.* LPS+Lep 100	ns
Control *vs.* LPS+IFN+Lep 100	****
LPS *vs.* LPS+IFN	****
LPS *vs.* Lep 10	ns
LPS *vs.* LPS+Lep 10	ns
LPS *vs.* LPS+IFN+Lep 10	****
LPS *vs.* Lep 100	ns
LPS *vs.* LPS+Lep 100	ns
LPS *vs.* LPS+IFN+Lep 100	**
LPS+IFN *vs.* Lep 10	****
LPS+IFN *vs.* LPS+Lep 10	****
LPS+IFN *vs.* LPS+IFN+Lep 10	****
LPS+IFN *vs.* Lep 100	****
LPS+IFN *vs.* LPS+Lep 100	****

LPS+IFN *vs.* LPS+IFN+Lep 100	****
Lep 10 *vs.* LPS+Lep 10	ns
Lep 10 *vs.* LPS+IFN+Lep 10	****
Lep 10 *vs.* Lep 100	ns
Lep 10 *vs.* LPS+Lep 100	ns
Lep 10 *vs.* LPS+IFN+Lep 100	****
LPS+Lep 10 *vs.* LPS+IFN+Lep 10	****
LPS+Lep 10 *vs.* Lep 100	ns
LPS+Lep 10 *vs.* LPS+Lep 100	ns
LPS+Lep 10 *vs.* LPS+IFN+Lep 100	***
LPS+IFN+Lep 10 *vs.* Lep 100	****
LPS+IFN+Lep 10 *vs.* LPS+Lep 100	****
LPS+IFN+Lep 10 *vs.* LPS+IFN+Lep 100	**
Lep 100 *vs.* LPS+Lep 100	ns
Lep 100 *vs.* LPS+IFN+Lep 100	****
LPS+Lep 100 *vs.* LPS+IFN+Lep 100	****

B: -LPS

Two way ANOVA: **** $p < 0.0001$

Tukey's multiple comparisons.

ns $p > 0.05$ * $p < 0.05$ ** $p < 0.01$ *** $p < 0.001$ **** $p < 0.0001$

**Comparison**	**Significance**
**G-CSF**	
N-PEM [Control] *vs.* N-PEM/ Leptin 20 ng/mL	ns
N-PEM [Control] *vs.* N-PEM/ Leptin 100 ng/mL	ns
N-PEM/ Leptin 20 ng/mL *vs.* N-PEM/ Leptin 100 ng/mL	ns
**IL-1a**	
N-PEM [Control] *vs.* N-PEM/ Leptin 20 ng/mL	ns
N-PEM [Control] *vs.* N-PEM/ Leptin 100 ng/mL	ns
N-PEM/ Leptin 20 ng/mL *vs.* N-PEM/ Leptin 100 ng/mL	ns
**IL-6**	
N-PEM [Control] *vs.* N-PEM/ Leptin 20 ng/mL	ns
N-PEM [Control] *vs.* N-PEM/ Leptin 100 ng/mL	ns
N-PEM/ Leptin 20 ng/mL *vs.* N-PEM/ Leptin 100 ng/mL	ns
**IP-10**	
N-PEM [Control] *vs.* N-PEM/ Leptin 20 ng/mL	ns
N-PEM [Control] *vs.* N-PEM/ Leptin 100 ng/mL	ns
N-PEM/ Leptin 20 ng/mL *vs.* N-PEM/ Leptin 100 ng/mL	ns
**KC**	
N-PEM [Control] *vs.* N-PEM/ Leptin 20 ng/mL	ns
N-PEM [Control] *vs.* N-PEM/ Leptin 100 ng/mL	ns
N-PEM/ Leptin 20 ng/mL *vs.* N-PEM/ Leptin 100 ng/mL	ns

**MCP-1**	
N-PEM [Control] *vs.* N-PEM/ Leptin 20 ng/mL	ns
N-PEM [Control] *vs.* N-PEM/ Leptin 100 ng/mL	ns
N-PEM/ Leptin 20 ng/mL *vs.* N-PEM/ Leptin 100 ng/mL	ns
**MIP-1a**	
N-PEM [Control] *vs.* N-PEM/ Leptin 20 ng/mL	ns
N-PEM [Control] *vs.* N-PEM/ Leptin 100 ng/mL	ns
N-PEM/ Leptin 20 ng/mL *vs.* N-PEM/ Leptin 100 ng/mL	ns
**MIP-1b**	
N-PEM [Control] *vs.* N-PEM/ Leptin 20 ng/mL	ns
N-PEM [Control] *vs.* N-PEM/ Leptin 100 ng/mL	ns
N-PEM/ Leptin 20 ng/mL *vs.* N-PEM/ Leptin 100 ng/mL	ns
**MIP-2**	
N-PEM [Control] *vs.* N-PEM/ Leptin 20 ng/mL	**
N-PEM [Control] *vs.* N-PEM/ Leptin 100 ng/mL	ns
N-PEM/ Leptin 20 ng/mL *vs.* N-PEM/ Leptin 100 ng/mL	ns
**VEGF**	
N-PEM [Control] *vs.* N-PEM/ Leptin 20 ng/mL	ns
N-PEM [Control] *vs.* N-PEM/ Leptin 100 ng/mL	ns
N-PEM/ Leptin 20 ng/mL *vs.* N-PEM/ Leptin 100 ng/mL	ns

B: +LPS

Two way ANOVA: **** $p < 0.0001$

Tukey's multiple comparisons.

ns $p > 0.05$ * $p < 0.05$ ** $p < 0.01$ *** $p < 0.001$ **** $p < 0.0001$

**Comparison**	**Significance**
**G-CSF**	
N-PEM [LPS] *vs.* N-PEM [LPS]/ Leptin 20 ng/mL	ns
N-PEM [LPS] *vs.* N-PEM [LPS]/ Leptin 100 ng/mL	ns
N-PEM [LPS]/ Leptin 20 ng/mL *vs.* N-PEM [LPS]/ Leptin 100 ng/mL	ns
**GM-CSF**	
N-PEM [LPS] *vs.* N-PEM [LPS]/ Leptin 20 ng/mL	ns
N-PEM [LPS] *vs.* N-PEM [LPS]/ Leptin 100 ng/mL	ns
N-PEM [LPS]/ Leptin 20 ng/mL *vs.* N-PEM [LPS]/ Leptin 100 ng/mL	ns
**IFN-g**	
N-PEM [LPS] *vs.* N-PEM [LPS]/ Leptin 20 ng/mL	ns
N-PEM [LPS] *vs.* N-PEM [LPS]/ Leptin 100 ng/mL	ns
N-PEM [LPS]/ Leptin 20 ng/mL *vs.* N-PEM [LPS]/ Leptin 100 ng/mL	ns
**IL-1a**	
N-PEM [LPS] *vs.* N-PEM [LPS]/ Leptin 20 ng/mL	ns
N-PEM [LPS] *vs.* N-PEM [LPS]/ Leptin 100 ng/mL	ns
N-PEM [LPS]/ Leptin 20 ng/mL *vs.* N-PEM [LPS]/ Leptin 100 ng/mL	ns

**IL-1b**	
N-PEM [LPS] *vs.* N-PEM [LPS]/ Leptin 20 ng/mL	ns
N-PEM [LPS] *vs.* N-PEM [LPS]/ Leptin 100 ng/mL	ns
N-PEM [LPS]/ Leptin 20 ng/mL *vs.* N-PEM [LPS]/ Leptin 100 ng/mL	ns
**IL-5**	
N-PEM [LPS] *vs.* N-PEM [LPS]/ Leptin 20 ng/mL	ns
N-PEM [LPS] *vs.* N-PEM [LPS]/ Leptin 100 ng/mL	ns
N-PEM [LPS]/ Leptin 20 ng/mL *vs.* N-PEM [LPS]/ Leptin 100 ng/mL	ns
**IL-6**	
N-PEM [LPS] *vs.* N-PEM [LPS]/ Leptin 20 ng/mL	*
N-PEM [LPS] *vs.* N-PEM [LPS]/ Leptin 100 ng/mL	***
N-PEM [LPS]/ Leptin 20 ng/mL *vs.* N-PEM [LPS]/ Leptin 100 ng/mL	ns
**IL-9**	
N-PEM [LPS] *vs.* N-PEM [LPS]/ Leptin 20 ng/mL	ns
N-PEM [LPS] *vs.* N-PEM [LPS]/ Leptin 100 ng/mL	ns
N-PEM [LPS]/ Leptin 20 ng/mL *vs.* N-PEM [LPS]/ Leptin 100 ng/mL	ns
**IL-10**	
N-PEM [LPS] *vs.* N-PEM [LPS]/ Leptin 20 ng/mL	ns
N-PEM [LPS] *vs.* N-PEM [LPS]/ Leptin 100 ng/mL	ns
N-PEM [LPS]/ Leptin 20 ng/mL *vs.* N-PEM [LPS]/ Leptin 100 ng/mL	ns
**IL-12 (p70)**	
N-PEM [LPS] *vs.* N-PEM [LPS]/ Leptin 20 ng/mL	ns
N-PEM [LPS] *vs.* N-PEM [LPS]/ Leptin 100 ng/mL	ns
N-PEM [LPS]/ Leptin 20 ng/mL *vs.* N-PEM [LPS]/ Leptin 100 ng/mL	ns
**IL-13**	
N-PEM [LPS] *vs.* N-PEM [LPS]/ Leptin 20 ng/mL	ns
N-PEM [LPS] *vs.* N-PEM [LPS]/ Leptin 100 ng/mL	ns
N-PEM [LPS]/ Leptin 20 ng/mL *vs.* N-PEM [LPS]/ Leptin 100 ng/mL	ns
**IL-15**	
N-PEM [LPS] *vs.* N-PEM [LPS]/ Leptin 20 ng/mL	ns
N-PEM [LPS] *vs.* N-PEM [LPS]/ Leptin 100 ng/mL	ns
N-PEM [LPS]/ Leptin 20 ng/mL *vs.* N-PEM [LPS]/ Leptin 100 ng/mL	ns
**IL-17**	
N-PEM [LPS] *vs.* N-PEM [LPS]/ Leptin 20 ng/mL	ns
N-PEM [LPS] *vs.* N-PEM [LPS]/ Leptin 100 ng/mL	ns
N-PEM [LPS]/ Leptin 20 ng/mL *vs.* N-PEM [LPS]/ Leptin 100 ng/mL	ns
**IP-10**	
N-PEM [LPS] *vs.* N-PEM [LPS]/ Leptin 20 ng/mL	ns
N-PEM [LPS] *vs.* N-PEM [LPS]/ Leptin 100 ng/mL	ns
N-PEM [LPS]/ Leptin 20 ng/mL *vs.* N-PEM [LPS]/ Leptin 100 ng/mL	ns
**LIF**	
N-PEM [LPS] *vs.* N-PEM [LPS]/ Leptin 20 ng/mL	ns
N-PEM [LPS] *vs.* N-PEM [LPS]/ Leptin 100 ng/mL	ns
N-PEM [LPS]/ Leptin 20 ng/mL *vs.* N-PEM [LPS]/ Leptin 100 ng/mL	ns
**LIX**	
N-PEM [LPS] *vs.* N-PEM [LPS]/ Leptin 20 ng/mL	ns

N-PEM [LPS] *vs.* N-PEM [LPS]/ Leptin 100 ng/mL	ns
N-PEM [LPS]/ Leptin 20 ng/mL *vs.* N-PEM [LPS]/ Leptin 100 ng/mL	ns
**MCP-1**	
N-PEM [LPS] *vs.* N-PEM [LPS]/ Leptin 20 ng/mL	ns
N-PEM [LPS] *vs.* N-PEM [LPS]/ Leptin 100 ng/mL	ns
N-PEM [LPS]/ Leptin 20 ng/mL *vs.* N-PEM [LPS]/ Leptin 100 ng/mL	ns
**M-CSF**	
N-PEM [LPS] *vs.* N-PEM [LPS]/ Leptin 20 ng/mL	ns
N-PEM [LPS] *vs.* N-PEM [LPS]/ Leptin 100 ng/mL	ns
N-PEM [LPS]/ Leptin 20 ng/mL *vs.* N-PEM [LPS]/ Leptin 100 ng/mL	ns
**MIG**	
N-PEM [LPS] *vs.* N-PEM [LPS]/ Leptin 20 ng/mL	ns
N-PEM [LPS] *vs.* N-PEM [LPS]/ Leptin 100 ng/mL	ns
N-PEM [LPS]/ Leptin 20 ng/mL *vs.* N-PEM [LPS]/ Leptin 100 ng/mL	ns
**MIP-1a**	
N-PEM [LPS] *vs.* N-PEM [LPS]/ Leptin 20 ng/mL	ns
N-PEM [LPS] *vs.* N-PEM [LPS]/ Leptin 100 ng/mL	ns
N-PEM [LPS]/ Leptin 20 ng/mL *vs.* N-PEM [LPS]/ Leptin 100 ng/mL	ns
**RANTES**	
N-PEM [LPS] *vs.* N-PEM [LPS]/ Leptin 20 ng/mL	ns
N-PEM [LPS] *vs.* N-PEM [LPS]/ Leptin 100 ng/mL	ns
N-PEM [LPS]/ Leptin 20 ng/mL *vs.* N-PEM [LPS]/ Leptin 100 ng/mL	ns
**TNF-a**	
N-PEM [LPS] *vs.* N-PEM [LPS]/ Leptin 20 ng/mL	ns
N-PEM [LPS] *vs.* N-PEM [LPS]/ Leptin 100 ng/mL	**
N-PEM [LPS]/ Leptin 20 ng/mL *vs.* N-PEM [LPS]/ Leptin 100 ng/mL	ns
**VEGF**	
N-PEM [LPS] *vs.* N-PEM [LPS]/ Leptin 20 ng/mL	ns
N-PEM [LPS] *vs.* N-PEM [LPS]/ Leptin 100 ng/mL	ns
N-PEM [LPS]/ Leptin 20 ng/mL *vs.* N-PEM [LPS]/ Leptin 100 ng/mL	ns

Supplementary Tables of Figure 9. Statistical analysis of *in vivo* experiments.

C

Two way ANOVA: **** $p < 0.0001$

Tukey's multiple comparisons.

ns $p > 0.05$ * $p < 0.05$ ** $p < 0.01$ *** $p < 0.001$ **** $p < 0.0001$

**Comparison**	**Significance**
**0**	
Lean (10% Fat Diet) *vs.* Resistant (33% Fat Diet)	ns
Lean (10% Fat Diet) *vs.* Overweight (33% Fat Diet)	ns
Lean (10% Fat Diet) *vs.* Obese (33% Fat Diet)	ns
Resistant (33% Fat Diet) *vs.* Overweight (33% Fat Diet)	ns

Resistant (33% Fat Diet) *vs.* Obese (33% Fat Diet)	ns
Overweight (33% Fat Diet) *vs.* Obese (33% Fat Diet)	ns
**1**	
Lean (10% Fat Diet) *vs.* Resistant (33% Fat Diet)	ns
Lean (10% Fat Diet) *vs.* Overweight (33% Fat Diet)	ns
Lean (10% Fat Diet) *vs.* Obese (33% Fat Diet)	ns
Resistant (33% Fat Diet) *vs.* Overweight (33% Fat Diet)	ns
Resistant (33% Fat Diet) *vs.* Obese (33% Fat Diet)	ns
Overweight (33% Fat Diet) *vs.* Obese (33% Fat Diet)	ns
**2**	
Lean (10% Fat Diet) *vs.* Resistant (33% Fat Diet)	ns
Lean (10% Fat Diet) *vs.* Overweight (33% Fat Diet)	ns
Lean (10% Fat Diet) *vs.* Obese (33% Fat Diet)	**
Resistant (33% Fat Diet) *vs.* Overweight (33% Fat Diet)	ns
Resistant (33% Fat Diet) *vs.* Obese (33% Fat Diet)	*
Overweight (33% Fat Diet) *vs.* Obese (33% Fat Diet)	ns
**3**	
Lean (10% Fat Diet) *vs.* Resistant (33% Fat Diet)	ns
Lean (10% Fat Diet) *vs.* Overweight (33% Fat Diet)	***
Lean (10% Fat Diet) *vs.* Obese (33% Fat Diet)	****
Resistant (33% Fat Diet) *vs.* Overweight (33% Fat Diet)	**
Resistant (33% Fat Diet) *vs.* Obese (33% Fat Diet)	****
Overweight (33% Fat Diet) *vs.* Obese (33% Fat Diet)	ns
**4**	
Lean (10% Fat Diet) *vs.* Resistant (33% Fat Diet)	ns
Lean (10% Fat Diet) *vs.* Overweight (33% Fat Diet)	***
Lean (10% Fat Diet) *vs.* Obese (33% Fat Diet)	****
Resistant (33% Fat Diet) *vs.* Overweight (33% Fat Diet)	*
Resistant (33% Fat Diet) *vs.* Obese (33% Fat Diet)	****
Overweight (33% Fat Diet) *vs.* Obese (33% Fat Diet)	*
**5**	
Lean (10% Fat Diet) *vs.* Resistant (33% Fat Diet)	ns
Lean (10% Fat Diet) *vs.* Overweight (33% Fat Diet)	****
Lean (10% Fat Diet) *vs.* Obese (33% Fat Diet)	****
Resistant (33% Fat Diet) *vs.* Overweight (33% Fat Diet)	**
Resistant (33% Fat Diet) *vs.* Obese (33% Fat Diet)	****
Overweight (33% Fat Diet) *vs.* Obese (33% Fat Diet)	ns
**6**	
Lean (10% Fat Diet) *vs.* Resistant (33% Fat Diet)	ns
Lean (10% Fat Diet) *vs.* Overweight (33% Fat Diet)	****
Lean (10% Fat Diet) *vs.* Obese (33% Fat Diet)	****
Resistant (33% Fat Diet) *vs.* Overweight (33% Fat Diet)	****
Resistant (33% Fat Diet) *vs.* Obese (33% Fat Diet)	****
Overweight (33% Fat Diet) *vs.* Obese (33% Fat Diet)	*
**7**	
Lean (10% Fat Diet) *vs.* Resistant (33% Fat Diet)	ns
Lean (10% Fat Diet) *vs.* Overweight (33% Fat Diet)	****
Lean (10% Fat Diet) *vs.* Obese (33% Fat Diet)	****
Resistant (33% Fat Diet) *vs.* Overweight (33% Fat Diet)	***

Resistant (33% Fat Diet) *vs.* Obese (33% Fat Diet)	****
Overweight (33% Fat Diet) *vs.* Obese (33% Fat Diet)	*
**8**	
Lean (10% Fat Diet) *vs.* Resistant (33% Fat Diet)	ns
Lean (10% Fat Diet) *vs.* Overweight (33% Fat Diet)	****
Lean (10% Fat Diet) *vs.* Obese (33% Fat Diet)	****
Resistant (33% Fat Diet) *vs.* Overweight (33% Fat Diet)	****
Resistant (33% Fat Diet) *vs.* Obese (33% Fat Diet)	****
Overweight (33% Fat Diet) *vs.* Obese (33% Fat Diet)	**

D

Two way ANOVA: ** $p < 0.01$

Tukey's multiple comparisons.

ns $p > 0.05$ * $p < 0.05$ ** $p < 0.01$ *** $p < 0.001$ **** $p < 0.0001$

**Comparison**	**Significance**
**0**	
Control-Lean *vs.* Control-Resistant	ns
Control-Lean *vs.* Control-OW	ns
Control-Lean *vs.* Control-OB	ns
Control-Resistant *vs.* Control-OW	ns
Control-Resistant *vs.* Control-OB	ns
Control-OW *vs.* Control-OB	ns
**1**	
Control-Lean *vs.* Control-Resistant	ns
Control-Lean *vs.* Control-OW	ns
Control-Lean *vs.* Control-OB	ns
Control-Resistant *vs.* Control-OW	ns
Control-Resistant *vs.* Control-OB	ns
Control-OW *vs.* Control-OB	ns
**2**	
Control-Lean *vs.* Control-Resistant	ns
Control-Lean *vs.* Control-OW	ns
Control-Lean *vs.* Control-OB	ns
Control-Resistant *vs.* Control-OW	ns
Control-Resistant *vs.* Control-OB	ns
Control-OW *vs.* Control-OB	ns
**3**	
Control-Lean *vs.* Control-Resistant	ns
Control-Lean *vs.* Control-OW	*
Control-Lean *vs.* Control-OB	ns
Control-Resistant *vs.* Control-OW	*
Control-Resistant *vs.* Control-OB	ns
Control-OW *vs.* Control-OB	ns
**4**	
Control-Lean *vs.* Control-Resistant	ns

Control-Lean *vs.* Control-OW	ns
Control-Lean *vs.* Control-OB	*
Control-Resistant *vs.* Control-OW	ns
Control-Resistant *vs.* Control-OB	ns
Control-OW *vs.* Control-OB	ns

<u>G</u>

Two way ANOVA: *** $p < 0.001$

Sidak's's multiple comparisons.

ns $p > 0.05$ * $p < 0.05$ ** $p < 0.01$ *** $p < 0.001$ **** $p < 0.0001$

**Comparison**	**Significance**
**Untreated - Treated**	
Lean	ns
Resistant	ns
Overweight	ns
Obese	**

Tukey's multiple comparisons.

ns $p > 0.05$ * $p < 0.05$ **$p < 0.01$ ***$p < 0.001$ ****$p < 0.0001$

**Comparison**	**Significance**
**Untreated**	
Lean *vs.* Resistant	ns
Lean *vs.* Overweight	ns
Lean *vs.* Obese	ns
Resistant *vs.* Overweight	ns
Resistant *vs.* Obese	*
Overweight *vs.* Obese	ns
**Treated**	
Lean *vs.* Resistant	ns
Lean *vs.* Overweight	ns
Lean *vs.* Obese	ns
Resistant *vs.* Overweight	ns
Resistant *vs.* Obese	ns
Overweight *vs.* Obese	ns

© 2015 by the authors; licensee MDPI, Basel, Switzerland. This article is an open access article distributed under the terms and conditions of the Creative Commons Attribution license (http://creativecommons.org/licenses/by/4.0/).
